# MicroRNA filters Hox temporal transcription noise to confer boundary formation in the spinal cord

**DOI:** 10.1038/ncomms14685

**Published:** 2017-03-24

**Authors:** Chung-Jung Li, Tian Hong, Ying-Tsen Tung, Ya-Ping Yen, Ho-Chiang Hsu, Ya-Lin Lu, Mien Chang, Qing Nie, Jun-An Chen

**Affiliations:** 1 Molecular and Cell Biology, Taiwan International Graduate Program, Academia Sinica and Graduate Institute of Life Sciences, National Defense Medical Center, Taipei 11529, Taiwan; 2 Institute of Molecular Biology, Academia Sinica, Taipei 11529, Taiwan; 3 Center for Complex Biological Systems, Department of Mathematics, University of California, Irvine, California 92697, USA; 4 Department of Developmental and Cell Biology, University of California, Irvine, California 92697, USA; 5 Institute of Biotechnology, College of Bio-Resources and Agriculture, National Taiwan University, Taipei 10672, Taiwan

## Abstract

The initial rostrocaudal patterning of the neural tube leads to differential expression of *Hox* genes that contribute to the specification of motor neuron (MN) subtype identity. Although several 3′ *Hox* mRNAs are expressed in progenitors in a noisy manner, these Hox proteins are not expressed in the progenitors and only become detectable in postmitotic MNs. MicroRNA biogenesis impairment leads to precocious expression and propagates the noise of Hoxa5 at the protein level, resulting in an imprecise Hoxa5-Hoxc8 boundary. Here we uncover, using *in silico* simulation, two feed-forward Hox-miRNA loops accounting for the precocious and noisy Hoxa5 expression, as well as an ill-defined boundary phenotype in *Dicer* mutants. Finally, we identify *mir-27* as a major regulator coordinating the temporal delay and spatial boundary of Hox protein expression. Our results provide a novel *trans* Hox-miRNA circuit filtering transcription noise and controlling the timing of protein expression to confer robust individual MN identity.

## Abstract (web summary)

In the spinal cord, some *Hox* genes are transcribed in progenitors while their proteins are only detected in differentiating postmitotic motor neurons. Here, the authors show that miRNAs (specifically *mir-27*) regulate post-transcriptional Hoxa5 expression in motor neurons.

In most bilateral animals, the axial identity along the rostrocaudal (RC) axis of the neural tube is defined by the homeobox (*Hox)* cluster genes, which encode an array of conserved homeodomain transcription factors. Mutations of these Hox proteins usually lead to homeotic transformation^1^,^2^,^3^. The expression of Hox genes along the RC axis of developing embryos is concordant with its 3′-to-5′ aligned direction within the Hox cluster that is usually referred to as ‘spatial collinearity' of Hox genes. In addition, Hox genes are activated one after the other sequentially in the 3′-to-5′ direction in a process described as ‘temporal collinearity.' Although, both spatial and temporal collinearity features are known to be highly conserved across bilaterians, the molecular details and the overall mechanism underlying the coordination of spatiotemporal collinearity of Hox genes is still obscure.

In addition to their well-known function in defining early axial identity, Hox cluster genes play critical roles in neural circuit formation by adopting cell-type specific programs that define the synaptic specificity of neuronal subtypes in the hindbrain and spinal cord^4^,^5^,^6^. The role of RC positional identity in neuronal specification has been carefully examined in the context of spinal motor neuron (MN) development, where there is a clear segregation of MN subtypes targeting specific muscles along the RC axis of the spinal cord. Gradients of retinoic acid (RA), fibroblast growth factor (FGF), and growth differentiation factor 11 (Gdf11) establish initial patterns of Hox gene expression in the early embryos^7^,^8^,^9^. Rostral RA primarily induces *Hox1* through *Hox5* genes, whereas FGF at the posterior tip induces *Hox6* through *Hox9* and Gdf11/FGF8 activate *Hox10* and *Hox11* genes. Hox proteins then interpret the extrinsic signals to define the individual neuronal identity by mutually exclusive expression. Yet, how opposing gradients (RA and FGF) cross-talk and how the spatial or temporal components of morphogen gradients coordinate to set up the precise boundary and neuronal subtype remain enigmatic.

Within Hoxc6^on^ limb-innervating motor neurons (LMC-MNs), mutually exclusive expression between Hox5 and Hoxc8 proteins further establish the boundary between molecularly-defined motor pools. Hox5 proteins (Hoxa5 and Hoxc5) are required to generate the motor pool that expresses the transcription factor Runx1 in the rostral LMC neurons, whereas Hoxc8 is required in the caudal LMC neurons to generate the motor pools that express the transcription factors Pea3 and Scip^10^,^11^. Although genetic evidence shows that Hox cluster genes are important to demarcate motor neuron subtype identity and synaptic connectivity, it remains unclear how Hox cluster genes coordinate to robustly define the individual neuronal subtype identity and whether additional critical factors are involved for Hox gene regulation.

In recent years, it has become clear that microRNA (miRNA) embedded within the Hox clusters is important to refine Hox genetic dynamics to ensure axial identity^1^,^12^,^13^,^14^. For example, *miR-10* resides in almost all taxa between *Hox4* and *5* paralogs and arose in early bilaterians, while *miR-196* is located between *Hox9* and *10* paralogs and is specific to vertebrates and urochordates. Genetic knockout or overexpression studies further indicate that Hox-embedded miRNAs are involved in regulating Hox gene expression at the post-transcriptional level^15^,^16^,^17^,^18^,^19^. Interestingly, while Hox genes are transcribed in spinal progenitors, many Hox proteins are only detectable in postmitotic MNs^7^,^20^. Several Hox transcripts are localized in broader domains than their corresponding proteins^21^,^22^, indicating that post-transcriptional regulation is involved in the refinement of the spatiotemporal Hox collinearity features reflected at transcriptional levels in the developing spinal cord. As we and others have demonstrated that miRNAs are critical in dorsoventral progenitor patterning, as well as motor neuron subtype diversification in the spinal cord^23^,^24^,^25^,^26^,^27^,^28^, here we aimed to test whether miRNAs might prevent precocious Hox protein expression until neurons differentiate and whether this regulation is functionally important.

In this study, we first verify that most 3′ *Hox* transcripts are activated upon RA activation in embryonic stem cell (ESC)-derived neuroepithelia. We find that the *Hoxa5* transcript displays robust but noisy transcription at motor neuron progenitors (pMNs), and conditional deletion of the miRNA biogenesis enzyme *Dicer* in neural progenitors leads to precocious and fluctuated expression of Hoxa5 protein, which results in an imprecise boundary between Hoxa5-Hoxc8 *in vitro* and *in vivo*. By taking advantage of *in silico* simulation of the Hox gene and miRNA network, we find that removing two critical feed-forward Hox-miRNA loops can recapitulate the precocious noisy Hoxa5 expression and rough boundary phenotype of *Dicer* mutants. Moreover, we identify *mir-27* as a major regulator governing temporal and spatial collinearity of Hox protein expression, which emphasizes the emerging role of miRNA in filtering protein expression noise and provides evidence that miRNA confers precision to protein expression, thereby controlling the developmental boundary and individual cell identity.

## Results

### Hox protein expression lags in spinal pMNs

**Figure 1:**
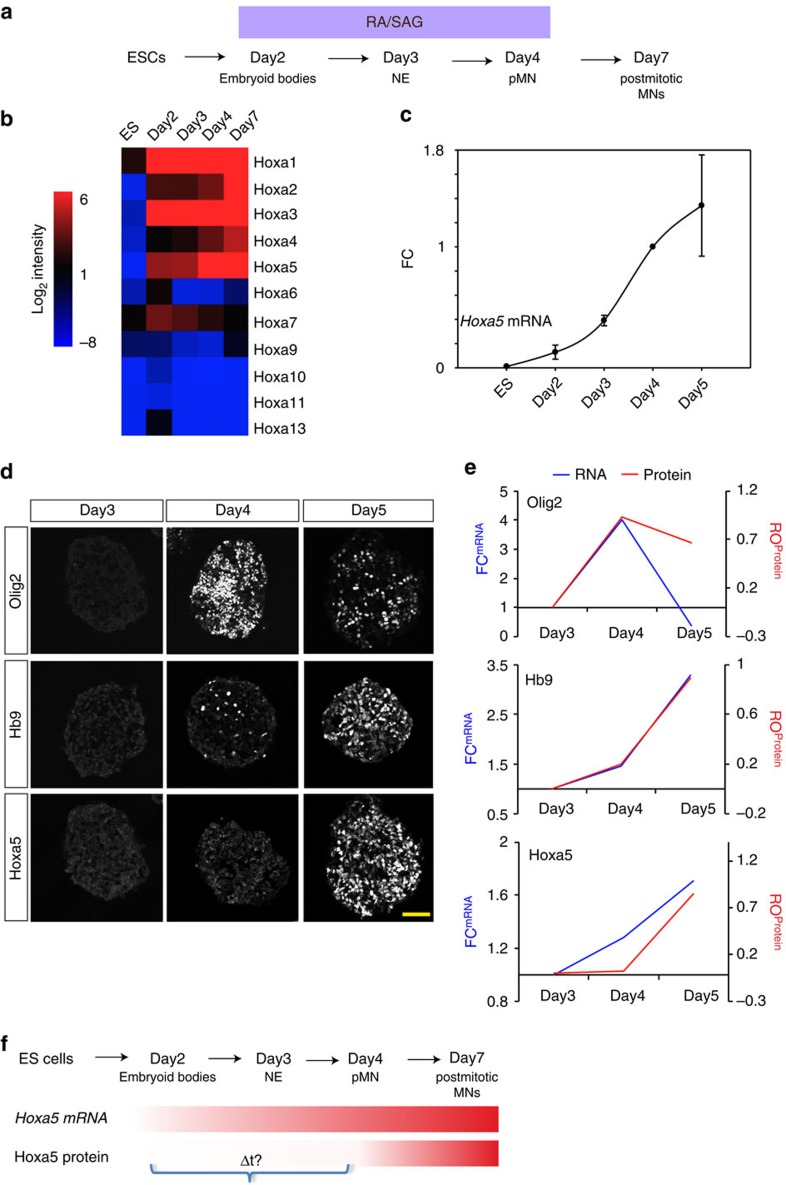
Temporal delay of Hox protein expression in postmitotic motor neurons. (**a**) Schematic illustration of protocol to generate spinal motor neurons from embryonic stem cells (ESCs). RA: retinoic acid. SAG: smoothened agonist. NE: neural epithelium. pMN: motor neuron progenitors. MNs: motor neurons. (**b**) Rapid induction of *Hoxa1*–*Hoxa5* gene expression upon RA/SAG induction. Heat map represents the intensity of gene expression along the *Hoxa* cluster at five time-points during motor neuron differentiation by Quantile analysis. (**c**) Quantitative PCR (qPCR) analysis of *Hoxa5* during MN differentiation. mRNA levels were normalized against Day4 pMN cells (mean±s.d., *n*=3 independent experiments). FC-fold change. (**d**) Immunodetection of Olig2, Hb9 and Hoxa5 in EBs under RA/SAG conditions. Scale bar represents 50 μm. (**e**,**f**) Comparisons of Olig2, Hb9 and Hoxa5 mRNA and protein expression by qPCR and immunostaining. mRNA/protein levels were normalized against Day 3 EBs (mean±s.d., *n*=3 independent experiments). Only Hoxa5 exhibits a significant delay of ∼72 h between gene and protein expression (**f**). FC-fold change, RO-ratio.

The pattern of Hox gene expression is established by a rostrocaudal gradient of morphogens and the mutually exclusive expression of Hox proteins in which their cross-repressive interactions further consolidate the individual motor neuron subtype identity^5^,^6^. While several Hox genes contain RA response elements within proximal enhancers and have their transcription directly activated by RA signal, many Hox proteins accumulate only in postmitotic MNs^7^,^29^. Furthermore, the mechanisms contributing to the time lag between Hox transcription and translation are still unknown. To dissect the significance underlying the delay between Hox transcription and translation, we first profiled global Hox gene expression using an *in vitro* ESC-derived motor neuron differentiation system (Fig. 1a; Supplementary Fig. 1a). Consistent with previous study^29^, most of the 3′ Hox genes were robustly induced upon RA treatment within 8 h (differentiation Day2), particularly those from the HoxA and HoxC clusters (Fig. 1b; Supplementary Table 1). As ESC-derived MNs subjected to RA/Smoothened Agonist (SAG) conditions acquired cervical/brachial Hoxa5^on^ identity^29^,^30^, we focused on confirming the temporal and spatial expression of the *Hoxa5* gene and protein by quantitative real-time PCR (qPCR) and immunostaining. In contrast to the robust induction of *Hoxa5* transcription in neuroepithelial embryoid bodies (EBs, Days 3–4), Hoxa5 protein only became detectable in postmitotic MNs (Day5) (Fig. 1c–f). Concomitantly, our *in situ* hybridization of the *Hoxa5* transcripts and immunostaining of Hoxa5 protein on the same cervical spinal section at E12.5 further confirmed the discrepancy between *Hoxa5* mRNA and protein expression in the progenitor ventricular zone (Supplementary Fig. 1b).

To reveal if the timing delay was simply a reflection of the sensitivity of the Hoxa5 antibody used in this study, we generated a ‘Tet ON' iHoxa5 ESC and induced exogenous *Hoxa5* mRNA expression with a linear concentration gradient of Dox (doxycycline), as the timing and level of expression of the inducible gene in this ESC can be accurately controlled by Dox (ref. 31) (Supplementary Fig. 2a). We then used qPCR to determine the corresponding Dox dosage for the comparable endogenous *Hoxa5* mRNA expression level in ESC-derived Day2 and 5 differentiated EBs. Given that exogenously-induced *Hoxa5* did not contain a 3′UTR, we could then systematically test the Hoxa5 mRNA/protein correlation and sensitivity in parallel between exogenous and endogenous *Hoxa5* (Supplementary Fig. 2b).

We tested several available Hoxa5 antibodies used in this study^10^ (Supplementary Fig. 2c and d). We observed robust and early Hoxa5^exo^ protein expression at a low Dox dosage (125 ng ml^−1^), while the corresponding [Dox] for *Hoxa5*^*endo*^ in Day2 EBs did not manifest detectable Hoxa5, as revealed by immunostaining. This result indicates that the Hoxa5 antibodies used in this study have high sensitivity, and the expression timing delay between *Hoxa5* mRNA and protein during MN differentiation is attributable to 3′UTR-mediated post-transcriptional repression *in vivo*. Altogether, we conclude that Hoxa5 has significantly delayed protein expression in the developing spinal cord.

### Strong heterogeneous *Hoxa5* mRNA expression in pMN

**Figure 2:**
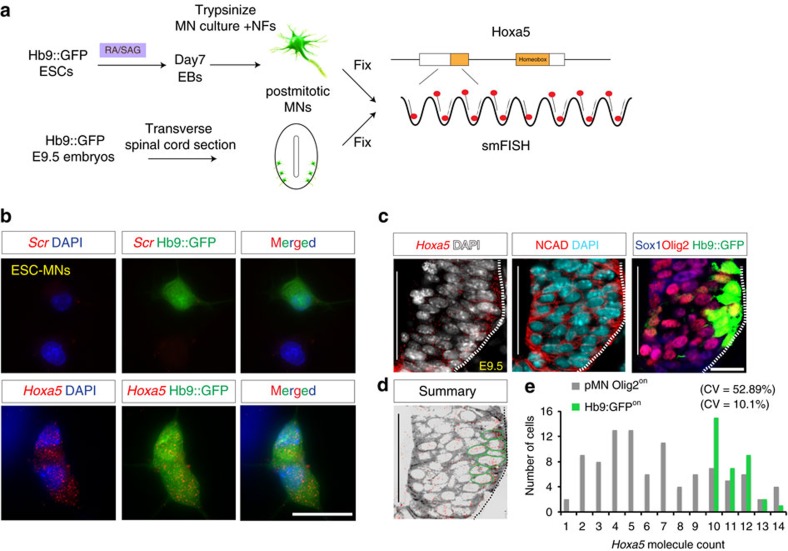
Strong heterogeneous expression levels of *Hoxa5* mRNAs in the pMNs. (**a**) Schematic illustration of single molecule RNA FISH (smRNA FISH) of *Hoxa5* transcripts *in vitro* and *in vivo*. (**b**,**c**) smRNA FISH of *Hoxa5* transcripts on *Hb9::GFP* ESC-derived motor neurons (**b**) or on sagittal sections (5–10 μm) of mouse spinal cord of E9.5 *Hb9::GFP* mouse embryos (**c**). Scr: scrambled sequence control probe. Scale bar represents 20 μm. (**d**,**e**) smRNA FISH signal quantifications show strong cellular variance of *Hoxa5* mRNA number in the pMNs (Olig2^on^, GFP^off^) compared to robust and steady *Hoxa5* levels in postmitotic MNs (GFP^on^). N-cadherin (NCAD) is used to demarcate cell boundaries and nuclei are counterstained with DAPI. CV: coefficient of variation.

Why does *Hoxa5* exhibit significant mRNA expression in the progenitors and execute delayed protein expression until the postmitotic stage? As transcription is relatively noisy in living organisms and stem cells^32^,^33^, we tested if *Hoxa5* transcription exhibits a strong heterogeneous pattern in the pMNs. To study this, we performed single molecule RNA FISH (smFISH) to visualize *Hoxa5* at single-cell resolution. We designed probes to recognize unique *Hoxa5* sequences to prevent cross-reaction with the conserved homeodomain region of other *Hox* genes (Fig. 2a). First, we tested the specificity of smFISH probes in ESC-derived MNs. Compared to a scramble (Scr) control probe that did not show signals, the *Hoxa5* probe set manifested strong punctate signals in Hb9::GFP^on^ MNs (Fig. 2b). To further corroborate the *in vitro* observation, we performed smFISH for *Hoxa5* in the spinal cord sections of E9.5 *Hb9::GFP* embryos. N-cadherin was used to outline the cell margin, whereas Olig2/Sox1 and Hb9::GFP were used to reflect pMNs and post-mitotic MNs on adjacent sections (Fig. 2c). Compared to the relative homogenous expression within postmitotic GFP^on^ MNs, strong cell-to-cell variation of *Hoxa5* transcripts in pMNs was observed (Fig. 2d and quantification in 2E, *n*=5 embryos). These results indicate that *Hoxa5* mRNAs exhibit strong cell-to-cell fluctuations in pMNs, so that pMNs might need a noise-filtering machinery to prevent precocious protein expression at this stage.

### *Dicer^−/−^* causes precocious and noisy Hoxa5 protein expression

Given that several studies have indicated that Hox genes can be either regulated post-transcriptionally^17^,^34^,^35^, we investigated whether miRNAs are required to regulate the timing of Hoxa5 protein expression. We first used conditional *Dicer* ESCs in which one or both *Dicer* alleles can be disrupted by 4-hydroxytamoxifen (4-OHT) treatment, and examined if the decreased levels of miRNAs affect the timing of Hoxa5 protein expression (Supplementary Fig. 3a). On Day4 of differentiation, control cells did not manifest Hoxa5 proteins in the EBs, whereas identical treatment of the *Dicer*^*−/−*^ ESC resulted in a significant increase in the percentage of progenitors expressing Hoxa5 (*n*=3 independent experiments; Supplementary Fig. 3b–d). Precocious Hoxa5 protein expression was not attributable to mRNA level, as qPCR revealed comparable *Hoxa5* in control and *Dicer*^*−/−*^ EBs (Supplementary Fig. 3e).

**Figure 3:**
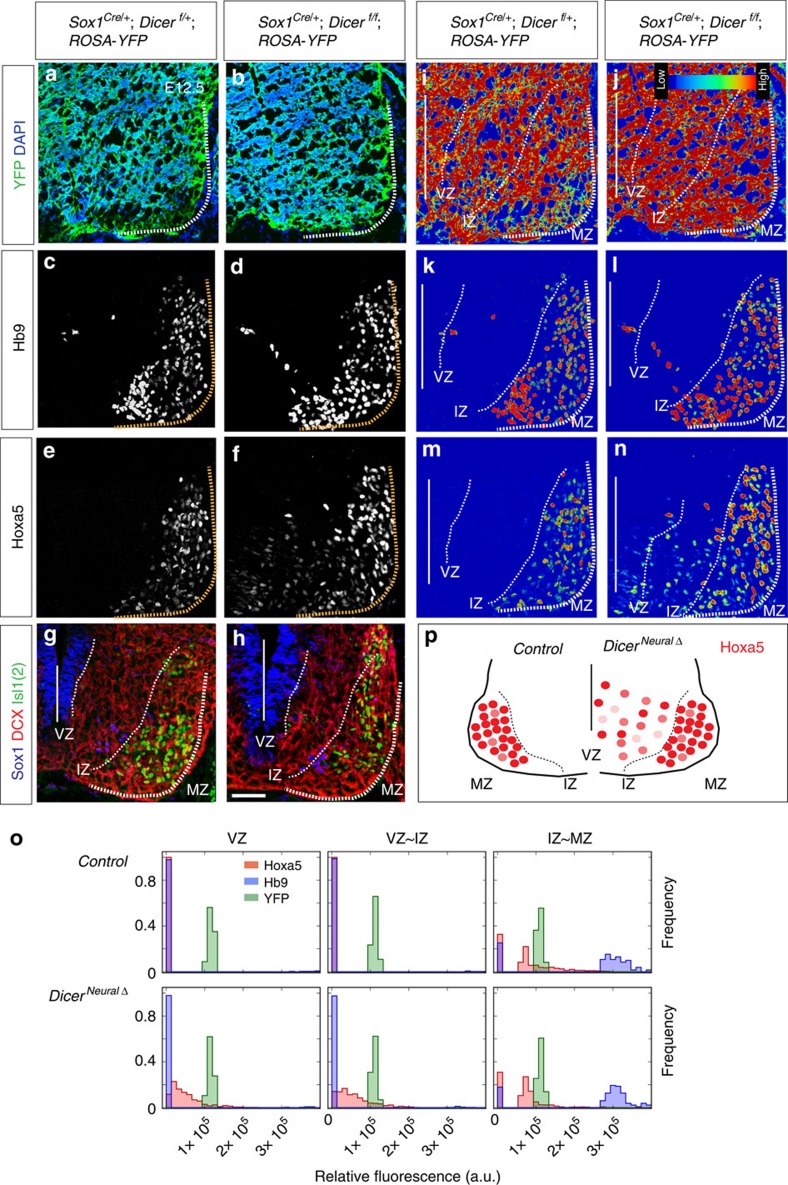
Dicer is critical to filter precocious noisy Hoxa5 protein expression in motor neuron progenitors. (**a**,**b**) *Sox1*^*Cre/+*^ driver mediates ∼100% floxed allele recombination in the spinal cord of their progenies, marked by YFP expression in *Sox1*^*Cre/+*^; *Dicer*^*f/+*^, *ROSA26-loxp-STOP-loxp-YFP* (*control*) or *Sox1*^*Cre/+*^; *Dicer*^*f/f*^, *ROSA26-loxp-STOP-loxp-YFP* (*Dicer*^*NeuralΔ*^) embryos. (**c**–**h**) Immunostaining at brachial spinal cord sections points to precocious Hoxa5 expression in the ventricular zone (VZ) and intermediate zone (IZ) of *Dicer*^*NeuralΔ*^ embryos, yet Hoxa5 is only expressed in the postmitotic marginal zone (MZ) in the control *Sox1*^*Cre/+*^*; Dicer*^*f/+*^ embryos. The VZ region is delineated by Sox1^on^; the IZ region is demarcated by DCX^on^, Sox1^off^, Isl1(2)^off^; and the MZ region is marked by Isl1(2)^on^, respectively. Scale bar represents 50 μm. (**i**–**n**) Heat map of protein expression level quantified by MetaXpress. (**o**) Histograms reflect the distributions of YFP^on^, Hb9^on^, and Hoxa5^on^ cells with variable intensity at brachial spinal cord in control and *Dicer*^*NeuralΔ*^ embryos (mean±s.d., *n*=4 embryos). (**p**) Summary of precocious and noisy Hoxa5 protein expression in the pMNs of *Dicer* KO embryos.

Notably, the precocious Hoxa5 expression in *Dicer*^*−/−*^ EBs exhibited stronger cell-to-cell variability than the controls (Supplementary Fig. 3d). To further verify this phenotype *in vivo*, we examined the expression patterns of Hox proteins in *Sox2-Cre*^*Tg/+*^*; Dicer*^*floxed*^ (epiblast deletion at E5.5, referred to as *Dicer*^*epiblastΔ*^) embryos. Despite the spina bifida phenotype observed at E8.5 in the *Dicer*^*epiblastΔ*^ embryos (Supplementary Fig. 4a–d), neural tubes still displayed robust neural patterning markers (Pax6^on^, Olig2^on^ and Nkx2.2^on^ in Supplementary Fig. 4e and f) and morphogen signalling pathways were unaffected (Shh^on^ and Raldh2^on^ in Supplementary Fig. 4g). However, Hoxa5 protein was precociously expressed in the neural tube of *Dicer*^*epiblastΔ*^ embryos (Supplementary Fig. 4h). To circumvent the morphogenesis defects of neural progenitors in the *Dicer*^*epiblastΔ*^ embryos, we alternatively used *Sox1*^*Cre/+*^*; Dicer*^*floxed*^ with a *ROSA26-loxp-STOP-loxp YFP* reporter (neuroepithelium deletion from E7.5, hereafter referred to as *Dicer*^*neuralΔ*^ embryos), in which the miRNA biogenesis pathway was only impaired in the central nervous system (Supplementary Fig. 4i and j; Fig. 3a,b). *Dicer*^*neuralΔ*^ embryos exhibited perinatal lethality and a shortened axis (Supplementary Fig. 4k). We verified that the recombination efficiency of *Sox1*^*Cre/+*^ in the spinal cord is nearly ∼100% (Fig. 3a,b, protein intensity is quantified and shown by heat map in Fig. 3i,j). This is consistent with previously published literature using the same *Sox1*^*Cre/+*^ line, and no mosaic feature of this Cre line was raised^36^.

Next, we used Sox1/Doublecortin (Dcx) /Isl1(2) staining to demarcate the progenitor (VZ), nascent MNs (VZ–IZ) and postmitotic (IZ–MZ) zones (Fig. 3g,h). Although the intensity of YFP^on^ cells was homogenously distributed in all regions in the spinal cord, Hoxa5 was manifested precociously and a strong fluctuating pattern in the VZ–IZ zone of *Dicer*^*neuralΔ*^ embryos was observed. Notably, postmitotic Hoxa5^on^ and Hb9^on^ cells displayed a relatively uniform level of expression in the MZ region from control and *Dicer*^*neuralΔ*^ embryos (Fig. 3c–f, protein intensity reflected by heat map in Fig. 3k–n, and distribution quantification is shown as a histogram in 3O, *n*=3 embryos). Therefore, these analyses indicate that Hoxa5 displays precocious and fluctuating protein expression in the pMNs of the *Dicer*^*neuralΔ*^ embryos.

Taken altogether, we suggest that Dicer/miRNA biogenesis places a delay on production of Hoxa5 protein, allowing reduction of *Hoxa5* transcription noise. As a result, the impairment of Dicer/miRNA biogenesis leads to precocious propagation of the noisy Hoxa5 protein in pMNs (Fig. 3p).

### *Dicer* deletion results in a distorted Hoxa5/Hoxc8 boundary

**Figure 4:**
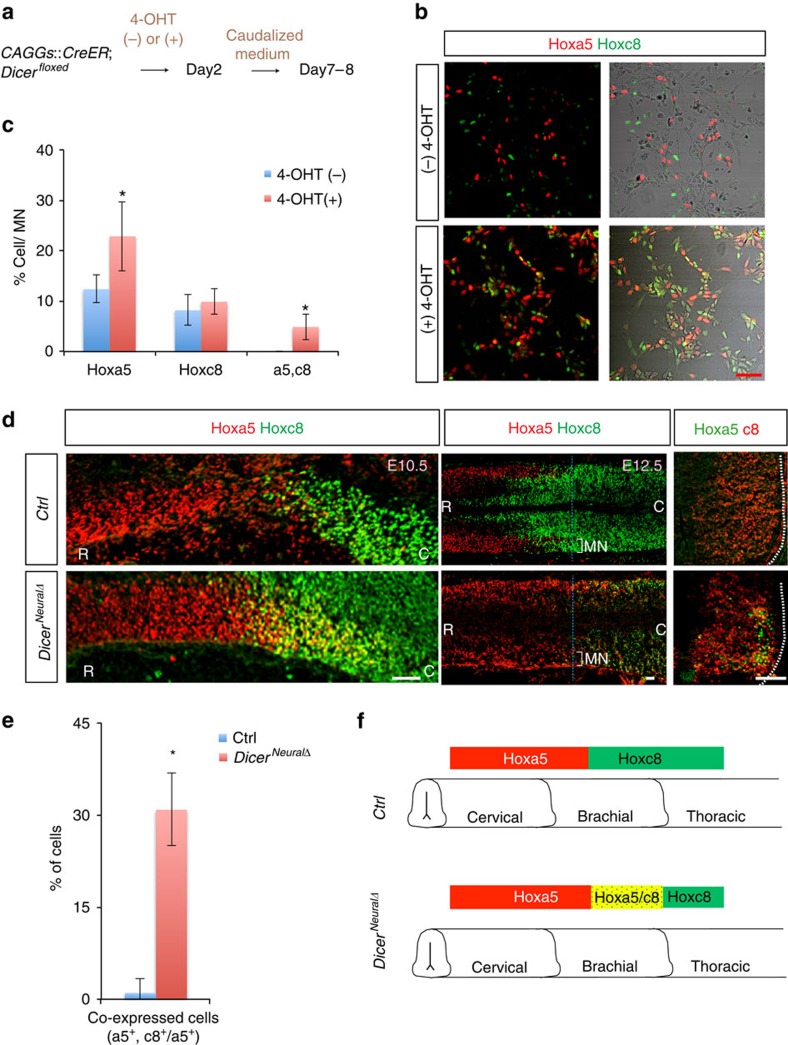
Precocious Hoxa5 expression in *Dicer* mutant leads to a noisy Hox5 –Hox8 boundary *in vitro* and *in vivo.* (**a**) *CAGGs:CreER; Dicer*^*floxed*^ ESC lines were treated with (+) or without (−) 4-hydroxytamoxifen (4-OHT) and differentiated with caudalized medium (see Experimental Procedures for details) on Day2. Postmitotic neurons were dissociated on Day5 and immunostaining was performed on Days 7 and 8 of differentiation. (**b**,**c**) Immunostaining of Day8 MN culture reveals that 4-OHT-treated cells display a significant increase in cells co-expressing Hoxa5 and Hoxc8, whereas non-treated cells manifest robust segregation of Hoxa5^on^ and Hoxc8^on^ cells (mean±s.d., *n*=3 independent experiments, *P<0.01). Scale bar represents 50 μm. (**d**) Immunostaining of spinal cord sections demonstrating noisy co-expressed Hoxa5/Hoxc8 in E10.5 (sagittal) and E12.5 (horizontal and transverse) *Sox1*^*Cre/+*^*; Dicer*^*f/f*^ (*Dicer*^*NeuralΔ*^) embryo sections, whereas mutually exclusive Hoxa5/Hoxc8 expression is present in the control embryos. Scale bar represents 50 μm. (**e**) Quantification of percentage of co-expressed Hoxa5^on^/Hoxc8^on^ cells against Hoxa5^on^ cells at brachial spinal cord (number of positive cells per 20 μm ventral spinal cord section) in control and *Dicer*^*NeuralΔ*^ embryos (mean±s.d., *n*=9 embryos, *P<0.01). (**f**) Summary of boundary shift of Hoxa5-Hoxc8 in *Dicer*^*NeuralΔ*^ EBs and embryos.

As a previous study demonstrated that Hoxa5 and Hoxc8 proteins define the rostrocaudal identity and position of motor pools^10^, we therefore tested whether the precocious and noisy Hoxa5 protein distribution leads to impairment of the boundary formation of postmitotic MNs. To examine whether the decreased levels of miRNAs affect the Hox boundary, 4-OHT-treated controls and conditional *Dicer*^*−/−*^ ESCs were exposed to caudalized medium on Day2 of differentiation (Fig. 4a). On Day7 of differentiation, controls exhibited comparable numbers of mutually-exclusive Hoxa5^on^ and Hoxc8^on^ cells in the EBs, yet *Dicer*^*−/−*^ EBs exhibited a significant increase of Hoxa5^on^ motor neurons. More importantly, ∼5–10% of motor neurons manifested co-expression of Hoxa5 and Hoxc8 (*n*=3 independent experiments; Fig. 4b,c).

To verify this phenotype *in vivo*, we examined the Hox expression in *Dicer*^*neuralΔ*^ embryos. While the reciprocal expression of Hoxa5 and Hoxc8 was maintained along the rostrocaudal axis in the Hox6^on^ LMC motor neurons in the control embryos, Hoxa5 was expanded caudally into Hoxc8^on^ territory, with a significant increase of Hoxa5/Hoxc8 co-expressing cells (*n*=9 embryos in Fig. 4d and quantification in 4e for transverse sections at E12.5).

Altogether, precocious Hoxa5 expression in pMNs leads to a less straight, more ill-defined Hoxa5-Hoxc8 boundary in postmitotic MNs. *Dicer*^*−/−*^ mutants displayed strong noisy patterns of Hox5 and Hox8 expression compared to the control, suggesting that miRNA biogenesis plays a critical role in buffering noise (Fig. 4f).

### Temporal and spatial modelling for Hox-miRNA interactions

**Figure 5:**
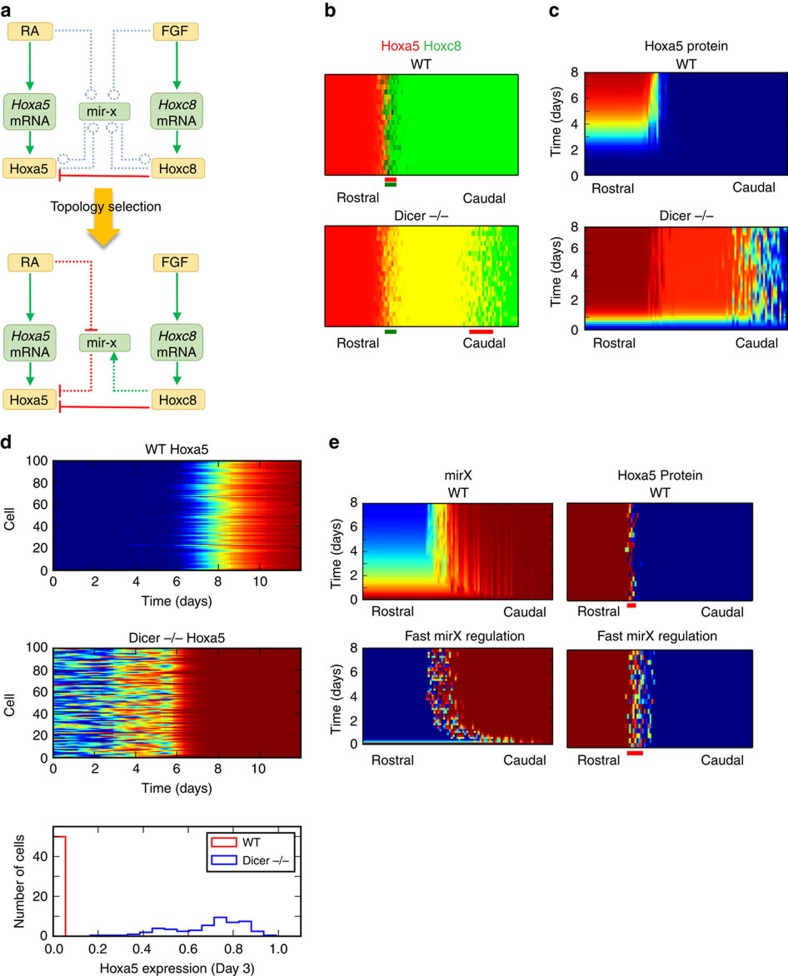
Mathematical simulation supports the critical Hox-miRNA circuitry. (**a**) Influence diagram for the basal model (upper) and predicted model (lower). (**b**) Spatial distribution of Hoxa5 (red) and Hoxc8 (green) at steady state for WT embryo (upper) and *Dicer*^*−/−*^ embryo (lower). Transitional zones for Hoxa5 (spatial domain in which 15–85% of cells of the same horizontal position are expressing Hoxa5) and Hoxc8 are marked as red and green bars, respectively. (**c**) Temporal dynamics of Hoxa5 protein during development along the rostral-caudal axis. (**d**) Temporal dynamics of Hoxa5 protein in a group of cells (WT or *Dicer*^*−/−*^) in response to RA stimulation only during ES-derived MN differentiation. Upper panels: heat maps showing the expression levels of Hoxa5. Lower panel: histogram showing the distributions of Hoxa5 protein in WT and *Dicer*^*−/−*^ cells at Day3. (**e**) Left: Temporal dynamics of mir-x during development along the rostral-caudal axis for normal (upper) and fast (lower) regulation speeds. Right: corresponding spatial distribution of Hoxa5 protein at steady state. Transition zones are marked as red bars.

Previous studies have shown that the dynamics of signalling networks is critical for robust formation of boundaries between adjacent domains in developing tissues^10^,^37^,^38^. To map the possible miRNA signalling network in regulating the timing of Hoxa5 protein expression and the robust formation of the Hoxa5/Hoxc8 boundary, we first built a mathematical model of cells expressing Hoxa5 and Hoxc8 in the developing spinal cord. The model describes each cell as a signalling network under the influence of various levels of RA and FGF depending on the position of the cell in the domain. We first built a preliminary network using known interactions in postmitotic MNs^5^,^6^. With this network and a set of basal parameters (see Supplementary Table 2 and Method), we incorporated a hypothetical miRNA (*mir-x*) and sampled parameter sets that represent 324 possible network topologies involving *mir-x* (Fig. 5a, upper panel). With this spatial model, we scored these topologies based on the robustness and accuracy of the Hoxa5/Hoxc8 boundaries in response to fluctuating morphogen signals (RA and FGF) (see Method). Interestingly, the top-scored topologies had three consensus interactions: (1) inhibition of *mir-x* by RA, (2) inhibition of Hoxa5 protein expression by *mir-x* and (3) activation of *mir-x* by Hoxc8 (Supplementary Fig. 5a and Fig. 5a, lower panel). Simulation with this predicted Hox-miRNA network recapitulated the spatial and temporal dynamics described in previous sections (Fig. 5b,c; Supplementary Fig. 5b). Moreover, the absence of *mir-x* in the model caused precocious expression of Hoxa5 protein and its rough boundary (Fig. 5b,c). The simulations were also consistent with the *Dicer*^*−/−*^ cells that have broader distributions of Hoxa5 protein than WT cells at the early stage of activation (Fig. 5d). This suggests that *mir-x* is involved in controlling both temporal fluctuations and spatial patterns of gene expression.

We noticed that the predicted Hox-miRNA network involves two coherent feed-forward loops via *mir-x* (Supplementary Fig. 5c). As this type of motif is known for its role in generating delayed responses and filtering noisy signal input^39^, we further examined the temporal behaviour of Hoxa5 protein expression in response to RA signal. Consistent with the experimental observations, our simulation showed that the presence of *mir-x* is essential for the delayed response of Hoxa5 protein (Supplementary Fig. 5d), suggesting a critical role of the coherent feed-forward loop in controlling temporal dynamics of Hoxa5. Since this delay is due to the relatively slow response of the indirect arm of this loop (RA-mir-x-Hoxa5, see Supplementary Fig. 5d), we examined whether accelerated responses via miRNA could cause precocious formation of the Hoxa5-expressing domain and roughness of its boundary. Indeed, the boundary of the Hoxa5-expressing domain became rough when the *mir-x*-mediated response was accelerated (Fig. 5e). This implies that the robustness of Hoxa5/Hoxc8 spatial patterning is influenced by the temporal dynamics of the miRNA signalling network. This model also suggests that early expression of *mir-x* occurs in the entire domain and its late expression is restricted to the Hoxc8 expression domain (Fig. 5e).

### Hoxa5 activities are modulated by *mir-27*

**Figure 6:**
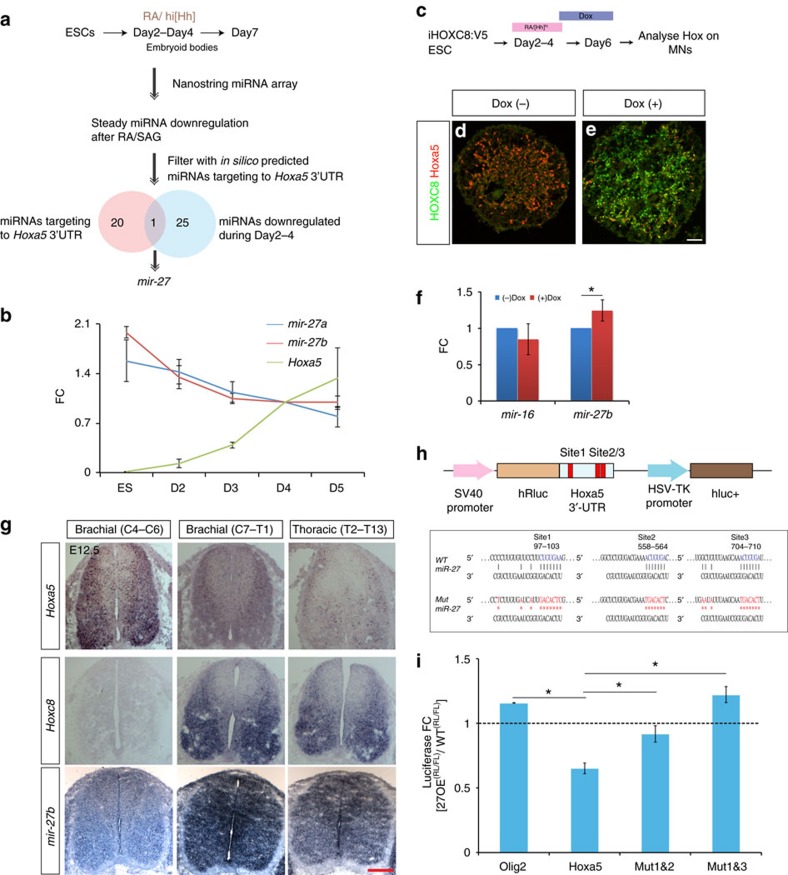
Systematic identification of *mir-27* as the primary candidate controlling Hoxa5 expression. (**a**) Strategy to identify miRNAs directly controlling Hoxa5 expression timing and the Hox boundary. (**b**) qPCR analysis of *mir-27a* and *mir-27b* during MN differentiation Day7. miRNA levels were normalized to Day4 (mean±s.d., *n*=3 independent experiments). FC-fold change. (**c**–**e**) Expression of Hoxa5 is repressed in inducible HOXC8 (iHOXC8) on Day 7-differentiated EBs under RA/SAG conditions and treated with Dox on Day 4. Effective repression of Hoxa5 upon HOXC8 induction is observed in (**e**). Scale bar represents 50 μm. (**f**) qPCR analysis reveals that *mir-27b* is maintained by HOXC8 (mean±s.d., *n*=3 independent experiments, *P<0.01). FC-fold change. (**g**) Expression of *Hoxa5*, *Hoxc8* and *mir-27b* examined by *in situ* hybridization on E12.5 spinal cord sections. *mir-27b* has prominent caudalized expression similar to *Hoxc8.* Scale bar represents 50 μm. (**h**) Luciferase reporters were constructed with either a control Hoxa5 3′UTR or the 3′UTR sequence in which the three potential target sites of *mir-27* were mutated (red) or deleted. (**i**) Co-expression of a luciferase construct with *mir-27b* in ESCs silences a reporter carrying intact *mir-27b* target sites, while *mir-27b* fails to silence Mut1&2 (site 1 and site 2 mutated) and Mut1&3 (site 1 and site 3 mutated) luciferase constructs (*n*=3 independent experiments, mean±s.d., *P*<0.01).

To test this model *in vivo*, we sought to identify which miRNA is responsible for the timing of Hoxa5 expression and refinement of the Hoxa5-Hoxc8 boundary in the spinal cord. We first profiled miRNA during *in vitro* ESC motor neuron differentiation using the NanoString platform and identified 20 miRNAs steadily downregulated by application of RA/SAG after differentiation (Fig. 6a; Supplementary Table 3). Out of these 20 miRNAs downregulated by RA, only one miRNA, *mir-27*, was predicted to target the 3′ UTR of *Hoxa5* mRNA across vertebrates by the target prediction algorithm TargetScan 6 (Fig. 6a). Independent qPCR analysis (*n*=3) confirmed that both expressions of *mir-27a* and *mir-27b* were gradually downregulated during ESC-derived MN differentiation (Fig. 6b). This is the opposite to the steady increase of Hoxa5 expression during MN differentiation. Therefore, complementary expression of *mir-27*/*Hoxa5* suggests that the delayed expression of Hoxa5 protein could be a reflection of strong *mir-27* expression in the pMNs.

As *mir-27a/b* have the same seed sequence and their expression profiles are similar, we only tested whether *mir-27b* could be induced by Hoxc8 in MNs. We generated a ‘Tet-ON' inducible HOXC8 ESC with a V5 tag and induced the expression of HOXC8 in pMN by doxycycline treatment on Day4 of differentiation, which resulted in efficient suppression of Hoxa5 expression (Fig. 6c–e; Supplementary Fig. 6a–e). Concomitantly, expression of *mir-27b* on Day6 was significantly induced upon HOXC8 induction, whereas ubiquitous *mir-16* was not affected in the postmitotic MNs (Fig. 6f, *n*=3 independent experiments). On the basis of these observations, we elected to examine the function of *mir-27* further in regulating the timing of Hoxa5 protein expression and boundary establishment.

### Dynamic *mir-27* expression along the RC axis of the spinal cord

To verify expression of *mir-27b* in the developing spinal cord, we performed whole mount *in situ* hybridization on E8.5 mouse embryos and revealed that *mir-27b* was highly expressed in all neuroepithelial tissues, yet was gradually caudalized and enriched in the brachial spinal cord at E9.5 (Supplementary Fig. 6f and g). This finding was consistent with our simulation results (Fig. 5e). At E12.5, *in situ* hybridization along the rostrocaudal axis of the spinal cord indicated that *mir-27b* was enriched in the caudal brachial (C7-T1) spinal region corresponding to the Hoxc8^on^ domain and was reduced in the rostral Hox5^on^ brachial region (Fig. 6g). To determine whether *Hoxa5* is a direct target of *mir-27*, we constructed a luciferase reporter containing the full length 3′ UTR of *Hoxa5* harbouring the three predicted *mir-27* target sites (Fig. 6h). We developed a ‘Tet-ON' inducible ESC line (i*Mir27b*), in which the primary *mir-27b* sequence was inserted into the 3′UTR of an inducible GFP construct^26^. Transfection of the luciferase construct with *mir-27b* overexpression upon doxycycline induction resulted in ∼40% reduction in luciferase activity (Fig. 6i), whereas an *Olig2* 3′UTR with no *mir-27* putative target sites was unaffected. The miRNA target prediction algorithms identified three potential binding sites for *mir-27*. We therefore mutated the three binding sites simultaneously or individually in *Hoxa5* 3′UTR reporter constructs (Fig. 6h), and constructs with combinations of two simultaneously-mutated putative *mir-27* binding sites were completely insensitive to *mir-27*-mediated silencing (Fig. 6i).

Taken altogether from the results of iHOXC8 analysis and *in situ* hybridization *in vivo*, as well as the luciferase assay, we suggest that *mir-27* is induced by Hoxc8 and is targeted to the 3′ UTR of *Hoxa5* directly.

### *Mir-23–27–24* DKO embryos phenocopy *Dicer*^*neuralΔ*^ embryos

**Figure 7:**
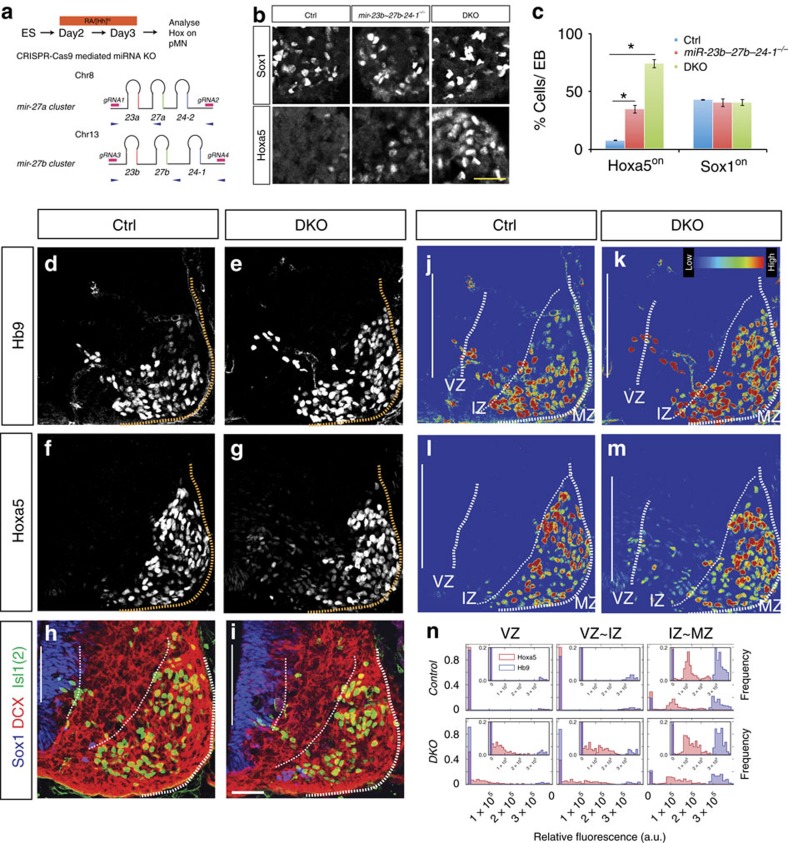
*miR-23–27–24* double KO embryos phenocopy *Dicer*^*neuralΔ*^ embryos. (**a**) Schematic illustration of genomic location and sequence alignment of mouse *miR-23–27–24* cluster miRNAs. Pink box indicates the gRNA targeting region. Blue arrows are shown to reveal primer sites for genotyping. (**b**,**c**) Expression of Hoxa5 and Sox1 in EBs from wild type (Ctrl) and *miR-23b–27b–24-1*^*−/−*^ or DKO ESCs cells. Deletion of *mir-27* results in precocious Hoxa5 expression, while control Ctrl cells have nearly no detectable Hoxa5^on^ cells. In contrast, both *miR-23b–27b–24-1*^*−/−*^ or DKO have no discernible effect on Sox1 expression (*n*=3 independent experiments, mean±s.d., *P*<0.01). Scale bar represents 50 μm. (**d**–**i**) Immunostaining at brachial spinal cord sections points to precocious Hoxa5 expression in the ventricular zone (VZ) and intermediate zone (IZ) in *miR-23–27–24* DKO embryos, yet Hoxa5 is only expressed in the postmitotic marginal zone (MZ) in the control wild type embryos. The VZ region is delineated by Sox1^on^; the IZ region is demarcated by DCX^on^, Sox1^off^, Isl1(2)^off^; and the MZ region is marked by Isl1(2)^on^, respectively. Scale bar represents 50 μm. (**j**–**m**) Heat map of protein expression level quantified by MetaXpress. (**n**) Histograms reflect the distributions of Hb9^on^, and Hoxa5^on^ cells with variable intensity at brachial spinal cord in control and *DKO* embryos (mean±s.d., *n*=3 embryos, *P<0.01).

To corroborate the role of *mir-27* in preventing Hoxa5 precocious protein expression and maintaining the precise boundary between Hoxa5-Hoxc8, we further examined Hoxa5 protein expression in CRISPR/Cas9-generated *mir-23–27–24* cluster double knockout (DKO) ESC-derived MNs^40^. Compared to wild type controls, Hoxa5 protein was precociously expressed in pMNs of *mir-23b–27b–24-1*^*−/−*^, and even more markedly in DKO, whereas Sox1^on^ cells were unaffected in Day3 progenitors (Fig. 7a–c). We then used the same gRNAs to generate *mir-23–27–24* cluster DKO embryos, verified the genotype, and tested the observed phenotype in *Dicer*^*neuralΔ*^ embryos. In the DKO embryos, we observed profound precocious and fluctuated Hoxa5 expression in the pMNs (E10.5), whereas the expression of Hb9 was unaffected (Fig. 7d–i, protein intensity reflected by heat map in Fig. 7j–m, and distribution quantification is shown as a histogram in Fig. 7n, *n*=3 embryos). Notably, this phenotype is similar to *Dicer*^*neuralΔ*^ embryos in Fig. 3. Thus, Hoxa5 displayed precocious and fluctuating protein expression in the pMNs of *mir-23–27–24* cluster DKO embryos.

### Characterization of *mir-27* in ESC–MNs and chicken embryos

**Figure 8:**
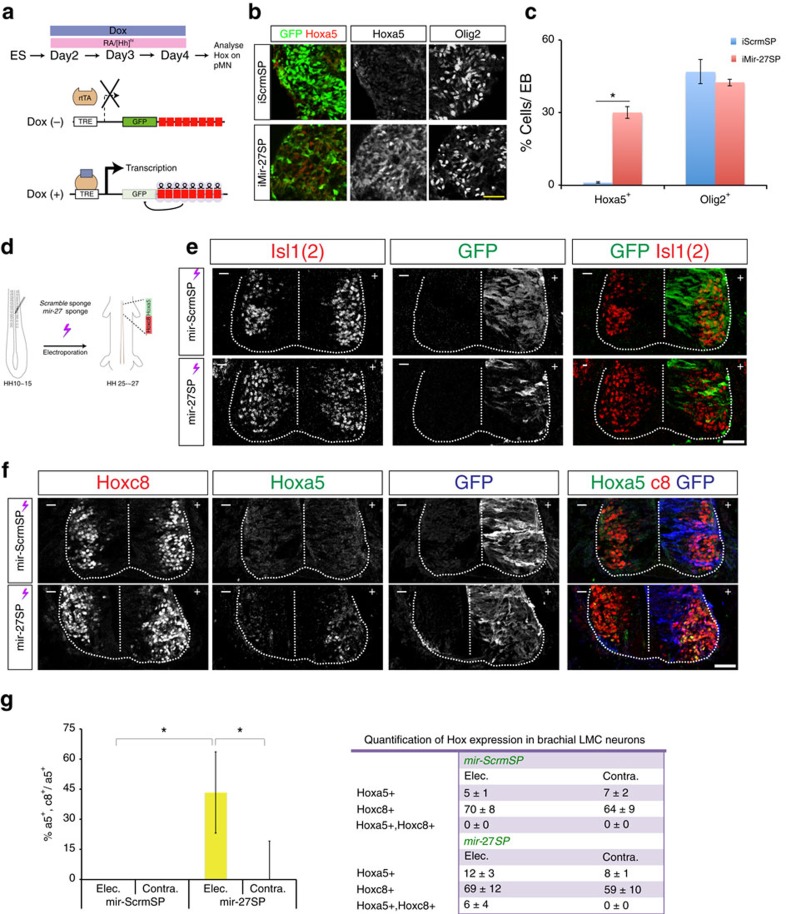
*mir-27* controls robustness of the Hoxa5-Hoc8 boundary. (**a**) Generation of inducible ESC lines expressing eight repetitive *mir-27b* sponge sequences inserted into the GFP 3′UTR. ESCs were differentiated under RA/SAG conditions with Dox treatment on Day 2 of differentiation. A scrambled sequence was inserted as a control. Scale bar represents 50 μm. (**b**,**c**) Expression of Hoxa5 and Olig2 in EBs from control (iScrmSP) and *mir-27b* sponge (iMir-27SP) cells. Induction of *mir-27b* sponge on Day 2 of differentiation under RA/SAG conditions led to precocious Hoxa5 expression, while control cells had no detectable Hoxa5. In contrast, induction of *mir-27bSP* had no discernible effect on Olig2 expression (*n*=3 independent experiments, mean±s.d., *P*<0.01). (**d**) Schematic illustration of the stages in performing *in ovo* electroporation in the embryonic spinal cord. (**e**,**f**) Immunostaining of brachial spinal cord sections demonstrates noisy co-expressed Hoxa5/Hoxc8 at electroporated side of *mir-27SP^elect^* spinal cords, whereas mutually exclusive Hoxa5/Hoxc8 expression is present in the control *mir-ScrmSP^elect^* spinal cords. Generic Isl1(2)^on^ MNs are unaffected. Scale bar represents 50 μm. (**g**) Quantification of percentage of co-expressed Hoxa5^on^Hoxc8^on^ cells against Hoxa5^on^ cells at brachial spinal cord (number of positive cells per 20 μm ventral spinal cord section) in *mir-ScrmSP^elect^* and *mir-27SP^elect^* chicken embryos (mean±s.d., *n*=3 embryos, *P<0.01).

To dissect if *mir-27* alone in the *mir-23–27–24* cluster can lead to the observed phenotype, we developed an *iMir27* sponge ESC (*iMir27SP*), in which eight sequence repeats complementary to *miR-27* were inserted into the 3′ UTR of GFP (Fig. 8a)^41^. To verify the dose requirement for stably saturating miRNA, we induced *iMir27SP* and a control 8 × repeated scramble sequence sponge (*iScrmSP*) with different doses of Dox. Both ESCs induced GFP in response to Dox dosages by the TRE promoter. In control *iScrmSP* cells, the number of positive GFP^on^ cells and the GFP mean fluorescence intensity increased proportionally with Dox concentration (Supplementary Fig. 6h). In *iMir27SP* ESCs, we observed a significantly lower GFP intensity at lower [Dox] (<500 ng ml^−1^), indicating that *mir-27* was regulating its endogenous targets.

To test whether *mir-27SP* recapitulated precocious Hoxa5 expression in *Dicer*^*−/−*^pMN cells, we applied 2 μg ml^−1^ Dox at Day2 of ESC-MN differentiation. We observed that the Hoxa5 protein level increased significantly in pMN cells (Day4) in *iMir27SP* cells, whereas Hoxa5 was not detected in the control *iScrmSP* cells (*n*=3 independent experiments, Fig. 8a–c). The precocious Hoxa5 protein expression in *mir-27SP* EBs was not a reflection of postmitotic fate, as Olig2^on^ progenitor cells were not affected (Fig. 8c).

To assess the effects of impairing *miR-27* activity on the Hox boundary in the embryonic spinal cord, we electroporated *mir-27SP* vectors into chick neural tubes as *mir-27* sequence and its targets sites upon *Hoxa5* are conserved in vertebrates (*mir-ScrmSP* separately as a control; Fig. 8d; Supplementary Fig. 6i). We then monitored the expression levels of GFP 2–3 days after electroporation. While Hoxa5-Hoxc8 segregated sharply in the control *mir-ScrmSP*^elect^ embryos, we observed a significant increase of co-expressed Hoxa5/Hoxc8 cells in the *mir-27SP*^elect^ embryos (Fig. 8f and quantification in 8g). The *miR-27* loss-of-function condition did not decrease the number of generic Isl1(2)^on^ postmitotic MNs (Fig. 8e), suggesting that the inhibitory effect of *miR-27* blockade is specific to the Hox boundary.

Taken altogether with the *in ovo*, *iMir27SP* EB, and *mir-27* DKO embryo studies, we conclude that *mir-27* controls the timing of Hox5 expression to confer the robustness of the Hoxa5-Hoxc8 boundary in the developing spinal cord (Supplementary Fig. 7).

## Discussion

Hox genes are well-known transcriptional regulators that elicit distinct developmental programs to orchestrate positional identities of cells and tissues. Recent studies have further revealed that Hox genes play multifaceted roles in controlling neuronal subtypes to confine synaptic specificity during development^5^,^6^. This indicates that Hox gene expression needs to be dynamically controlled at several levels to ensure proper axis formation and to elicit cell diversity in embryos. Here, using *in vitro, in silico* and *in vivo* approaches, we tested whether delayed Hoxa5 protein expression is controlled by miRNA and if this phenomenon is important for spatial boundary establishment.

Our results provide insights into the functional significance of the temporal delay in Hox protein expression. Before neural tube closure, morphogens first provide positional information for spatial patterns of gene expression during development. The retinoic acid morphogen signal leads to activation and binding of retinoic acid receptors (RARs) to the Hox1 through Hox5 chromatin domains, followed by a rapid domain-wide removal of H3K27me3 and acquisition of cervical spinal identity^29^,^42^. Interestingly, our single molecule FISH revealed that the *Hoxa5* transcript fluctuates in the pMNs, indicating that stochastic effects such as local fluctuations in morphogen concentration and noise in signal transduction make it difficult for pMNs to respond to their positional cues with sufficient fidelity to enable sharp boundary formation between gene expression domains^37^,^38^,^43^,^44^. In future experiments that adopt a single-cell approach to analyse pMNs from embryos, it will be interesting to examine the global Hox pattern at single-cell level and to see if this pattern is a universal phenomenon.

Previous studies have revealed that transcriptome-wide noise controls lineage choices in mammalian stem cells or progenitor cells, and cell-to-cell variability in the progenitors could be important in steering lineage choices of progenitors^33^,^45^,^46^. The slowly fluctuating transcriptome that is distinct from one progenitor cell to the next may govern the reversible, stochastic priming of multipotent progenitor cells in cell fate decisions, consistent with our previous study showing that the early spinal progenitors are malleable. To maintain the plasticity of progenitors, pMNs must act in concert and apply a refinement mechanism to ensure the robust output of motor neuron subtypes in adopting a given postmitotic cell fate in an ‘all-or-none' manner. Using mouse genetics and systems modelling, we have further shown that delaying the expression of Hox transcripts by miRNA and regulating the spatially and temporally varying gradient of RA signal through RA-Hox-miRNA logic can confer the robustness and reliability of motor neuron subtype diversification. This agrees with emerging evidence emphasizing that miRNA might function to canalize genetic programs, filter transcription noise and confer robustness and accuracy to gene expression. Our results further underscore that the precise timing of Hox gene activation is functionally important because the experimental conditions resulting in premature or delayed Hox gene activation have been shown to produce phenotypic alterations^47^,^48^,^49^,^50^.

Why do embryos adopt a delayed protein expression system controlled by Hox-miRNA logic? Studies in mouse and fly embryos demonstrate that Hox-regulating miRNAs are encoded within the Hox clusters. This genomic arrangement might provide an effective *cis* mechanism to ensure the posterior prevalence of Hox genes^12^. However, the *cis* embedded miRNAs in the Hox cluster are not expressed in the pMNs, and we therefore ruled out their potential role to regulate the delayed Hox protein expression in our system. Given that several lncRNA have been proven to act in *trans* to regulate the Hox epigenetic landscape^14^,^51^,^52^, our study here further provides evidence that *trans* miRNA (*mir-27*) outside the Hox cluster participates in the Hox network to confine the timing and robustness of Hox gene expression.

Our study has uncovered a novel role of miRNA in the formation of a robust and sharp boundary between two cell types by regulating the timing of Hox expression. Similar to DV progenitors exposed to Shh signals emanating from the notochord, Hox transcripts are induced according to the activity of RA and FGF. At this stage, progenitors responding to RA might still fluctuate, and the expression of *Hox* mRNA exhibits both temporal and cell-to-cell variability^37^,^43^. Translation of fluctuating transcripts at this time would propagate the noise, leading to strong stochastic variability. Due to slow inhibitory dynamics of RA on *mir-27*, fluctuating RA is unlikely to exert sustained inhibition on *mir-27*. Therefore, the coexistence of *mir-27* with *Hox* mRNA at this stage can prevent precocious Hoxa5 protein expression. At the nascent postmitotic stage, most MNs turn on *Hoxa5* expression more synchronously, and RA signalling stably inhibited *mir-27*, allowing robust nascent Hoxa5 protein expression. Consequently, Hoxc8 further maintains the expression of *mir-27* to generate a coherent feed-forward inhibition pathway to maintain the mutually exclusive Hoxa5-Hoxc8 sharp boundary^10^. This is similar to our previous study showing Olig2/Irx3/*mir-17-3p* constitutes a feed-forward circuit to carve the p2/pMN boundary^28^. We speculate that the relationship between the miRNA-induced protein expression delay and the robust boundary formation could be a general design principle for other miRNA circuits. As *cis*-embedded Hox miRNAs have been shown to ensure posterior prevalence during embryonic development, it will be equally interesting to see how *cis* and *trans* miRNAs together orchestrate Hox activity and provoke neuronal diversity along the RC axis of the spinal cord.

As coherent feed-forward loops are known to cause delayed responses and help to protect the system against brief fluctuations of signals in bacteria^53^, our mathematical model demonstrates that this network motif is critical for development of mammalian tissues by delaying the cellular response to morphogens, indicating its ubiquitous roles in biological systems. This control mechanism is distinct from the well-known bistable system that also confers robustness against morphogen fluctuations^38^, or the noise-driven sharpening of the boundary that depends upon bistability^43^. In the developing spinal cord, several previous studies and our inducible Hoxa5 ESC system revealed that Hoxa5 does not inhibit Hoxc8 (refs 5, 10). However, the expression of Hox transcription factors is generally controlled by selective, cross-repressive interactions that occur both rostrocaudally and within segments of the spinal cord^10^. As a consequence, minor fluctuations in starting Hox conditions within individual MNs will result in a ‘winner-take-all' extinction of expression of one or the other of two opponent Hox proteins on a largely stochastic basis. It is unclear whether Hoxa5-Hoxc8 asymmetric interactions might have a certain unexplored evolutionary advantage or some unknown positive feedback to facilitate this stochastic decision. It would be interesting to explore the possibilities in future studies. It is also possible that multiple mechanisms might contribute to the sharpening of the Hoxa5-Hoxc8 boundary. Future studies are warranted to explore the possible existence of positive feedback loops, which are essential to the creation of bistability in this system.

## Methods

### Mouse ES cell culture and differentiation

*Hb9::GFP*; *conditional Dicer floxed*, and *miR-23–27–24* single and double KO ESCs (gift from Yue Huang, PUMC China) were cultured and differentiated into spinal motor neurons^26^,^54^. In some cases, caudal LMC neuron differentiation was acquired by including 100 ng ml^−1^ bFGF together with reduced concentrations of RA and SAG at Day2 of differentiation^28^,^29^.

### Immunocytochemistry

Commercially available primary antibodies used in this study include: rabbit anti-HOXA5 (1/2,000, Sigma-Aldrich, HPA029319), HOXC5 (1/2,000, Sigma-Aldrich, HPA026794), HOXC8 (1/2,000, Sigma-Aldrich, HPA028911), guinea pig or rabbit anti-Hoxa5 (gifts from J Dasen and TM Jessell, and made in house), Hoxc8, Olig2, Pax6, Raldh2, Nkx2.2, Shh, guinea pig or rabbit anti-Hoxc6 and Hoxc9 (gifts from TM Jessell). Mouse monoclonal anti-Isl1(2), Hb9, and Hoxc8 were purchased from DSHB. Alexa488-, Cy3- and Cy5-conjugated secondary antibodies were obtained from either Invitrogen or Jackson Immunoresearch.

### miRNA *in situ* hybridization

Sections were fixed in 4% paraformaldehyde and acetylated in acetic anhydride/triethanolamine, followed by washes in PBS. Proteinase K treatment was skipped for post-immunostaining. Sections were pre-hybridized in hybridization solution (50% formamide, 5 × SSC, 0.5 mg ml^−1^ yeast tRNA, 1 × Denhardt's solution) at room temperature, then hybridized with 3′-DIG or FITC-labeled LNA probes (3 pmol) (LNA miRCURY probe; Exiqon) at 25 °C below the predicted Tm value. After post-hybridization washes in 0.2 × SSC at 55 °C, the *in situ* hybridization signals were detected using the NBT/BCIP (Roche) or Tyramide Signal Amplification system (Perkin-Elmer) according to the manufacturer's instructions. Slides were mounted in Aqua-Poly/Mount (Polysciences, Inc) and analysed with a Zeiss LSM510/710 Meta confocal microscope.

### Single molecule FISH

Embryos from various developmental stages (E9.5–E10.5) were obtained from timed matings of *Hb9::GFP* mice, and detection of a mating plug was counted as embryonic day 0.5 (E0.5). Embryos were dissected out, fixed in 4% paraformaldehyde in PBS for 2 h, and balanced in 30% sucrose after several washes. Fixed embryos were then embedded in OCT compound (Tissue-Tek), frozen in dry ice and stored at −80 °C until use. Spinal sections (5–10 μm) were made with a CM 1950 cryostat (Leica) and immediately placed on slides. For RNA FISH, sections on slides and cultured cells on coverslips were refixed in 4% paraformaldehyde for 10 min at room temperature, permeabilized for 5 min on ice in PBS with 0.5% Triton X-100, then rinsed in 70% EtOH for subsequent RNA FISH. Slides and coverslips were kept in 70% EtOH at 4 °C until staining. Sections were then washed in wash buffer (10% deionized formamide in 2 × SSC) for 5 min and incubated in a dark room at 37 °C for at least 4 h with 1 μl of probe stock solution and 100 μl of hybridization buffer (1 g dextran sulfate, 1 ml 20 × SSC, 1 ml deionized formamide). Hoxa5 and control smFISH probes were purchased from Stellaris. Images were captured with a Delta Vision microscopy system and quantified by Image J. N-cadherin was used to outline the cell margins; Olig2 and Hb9 were used to reflect pMNs and postmitotic MNs. The coefficient of variation was calculated from motor neuron progenitor and postmitotic Hb9::GFP^on^ regions from five embryos.

### Mouse crosses and *in vivo* studies

Conditional neural epithelium-knockout mice were generated by crossing *Sox1*^*Cre/+*^ mice^36^ (a kind gift from Shin-Ichi Nishikawa in RIKEN CDB) or *Sox2::Cre* (ref. 55) mice with *Dicer*^*loxp/loxp*^ (ref. 56) to generate the *Sox1*^*Cre/+*^*; Dicer*^*loxp/WT*^ strain*. Sox1*^*Cre/+*^*; Dicer*^*loxp/WT*^ mice were then mated with *Dicer*^*loxp/loxp*^ for experimental analysis. *miR-23–27–24* single and double KO embryos were made by a CRISPR-Cas9 mediated approach (IMB transgenic core). These mice were backcrossed to mice with a C57BL/6 background for eight generations before use. Mice were mated at age of 8–12 weeks and the embryo stage was estimated as E0.5 when copulation plug was observed. Embryos were analysed between E8.5 and 13.5. All of the live animals were kept in an SPF animal facility, approved and overseen by IACUC Academia Sinica.

### Analysis of Hoxa5 expression in *Dicer* mutants

The expression of Hoxa5 in control and *Dicer* mutants was imaged using a confocal system. The intensity of Hoxa5 protein was analysed by MetaXpress (Multi Wavelength Cell Scoring module, MWCS). The MWCS module can be used to analyse cells imaged in 1–7 wavelengths. Immunostaining of Sox1/Doublecortin (Dcx) /Isl1(2) were used to demarcate the progenitor (VZ), nascent MNs (VZ−IZ) and postmitotic (IZ–MZ) zone. We set up four individual wavelengths for DAPI, YFP, Hb9 and/or Hoxa5 staining, thereby getting DAPI^on^ and YFP^on^ cells; DAPI^on^ and Hb9^on^ cells; DAPI^on^ and Hoxa5^on^ cells. The results were quantified by YFP, Hb9, Hoxa5 pixel intensity for cells in VZ, VZ–IZ and IZ–MZ regions. The pixel intensities were plotted as histogram distributions (*N*=3 embryos from controls or KOs).

To define the threshold, we used the following formula:

Whole cell signal=sum of the intensity of the pixels for one cell.

Background signal=average signal per pixel outside of the spinal cord.

Whole cell signal corrected=Whole cell signal-Background signal.

Integrated Morphometry Analysis (IMA) representations were performed using the Metamorph Software. Briefly, for each IMA, we used 8 colour hues with 32 intensities ranked from maximum (red) to minimum (blue). The maximum and minimum values were calibrated and are indicated on each figure. The colour intensities displayed for each hue were determined automatically by the software and are reflected by histogram.

### Quantitative real time PCR

ESCs or embryoid bodies were harvested for total RNA isolation by the mirVana kit (Ambion). For mRNA analysis, 20 ng of total RNA from each sample was reverse transcribed with Superscript III (Invitrogen). One-tenth of the reverse transcription reaction was used for subsequent qRT-PCRs, which were performed in duplicate with at least three independent experimental samples on a LightCycler480 Real Time PCR instrument (Roche) using SYBR Green PCR mix (Roche) for each gene of interest and *Gapdh* was used as a control for normalization.

For miRNA analysis, 20 ng of total RNA was reverse transcribed with a miRNA-specific primer from TaqMan MicroRNA Assays (Life Technology). A ubiquitous small nucleolar RNA, sno202 or sno234, was used as the endogenous control. Each qRT-PCR was performed in duplicate or triplicate per sample with at least three different experimental samples.

### Generation of inducible ‘Tet-ON' ESCs

Human HOXC8 and mouse Hoxa5 cDNAs were directionally inserted into pENTR/D-TOPO vector (Life Technology) following manufacturer instructions. Primary miRNA sequence or repetitive miRNA sponge sequence was synthesized and cloned into the 3′UTR of p2Lox-GFP. Inducible lines were generated by treating the recipient ESCs for 16 h with Dox to induce Cre, followed by electroporation of p2Lox-HOXC8:V5/Hoxa5/miRNA OE/miRNA SP plasmids. After G418 selection, individual resistant clones were picked and characterized. After 10–15 days of selection, clones were expanded. Details of primer and miRNA sequences are listed in Supplementary Table 4.

Inducible miRNA overexpression and sponge ESCs were cloned into the 3′UTR of the p2Lox-GFP construct, and the same procedure as described above was followed to generate stable ESC clones.

### Luciferase reporter assay

*Hoxa5* 3′ UTRs were individually cloned into the psiCHECK-2 vector (Promega). *iMir-27OE or iMir-27SP* cells were plated at a density of 8 × 10^3^ per well (96-well plate), expanded for 20 h and transfected with 150 ng of reporter plasmids using 0.8 μl PLUS Reagent and 0.4 μl Lipofectamine LTX Reagent (Life Technology). Cells were lysed 24 h later and processed for luciferase assay using the Dual-Luciferase Reporter Assay System (Promega). Luciferase activity was measured using the Enspire Multimode Plate Reader (Perkin Elmer).

### *In ovo* electroporation

Neural tube electroporation of microRNA sponge constructs was performed on stage 10–15 (HH10–15) chick embryos. For misexpression of mir-sponge to decoy endogenous *mir-27* activity, plasmids were titrated (typically 1–4 μg μl^−1^ *CMV-GFP* vector). Electroporation efficiency was assayed by GFP expression in the spinal cord. In each experiment, ∼50 embryos were electroporated, with a survival efficiency of ∼20%, such that each set of results reflects an analysis of ∼10 manipulated embryos. Electroporation efficiencies in individual embryos ranged from 30 to 80% of LMC neurons at the segmental level under analysis, and we report results derived from embryos with >50% efficiency.

### Statistics

Statistical analysis was performed using Student's *t* test as all quantifications in the control and experimental sets are in similar sample sizes. All experiments were performed in parallel with both experimental and control genotypes. Error bars indicate s.d.

### Framework of mathematical model

To describe the system mathematically, we used a generic form of ordinary differential equations (ODEs) suitable for describing both gene expression and molecular interaction networks^57^,^58^. Each ODE system in the model has the form:


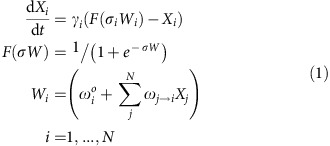


Here, *X*_*i*_ is the activity or concentration of protein *i*. On a time scale=1/*γ*_*i*_, *X*_*i*_ (t) relaxes toward a value determined by the sigmoidal function, *F*, which has a steepness set by *σ*. The basal value of *F*, in the absence of any influencing factors, is determined by 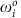. The coefficients *ω*_*j*→*i*_ determine the influence of protein *j* on protein *i*. *N* is the total number of proteins in the network. All variables and parameters are dimensionless. One time unit in the simulations corresponds to ∼1 day.

To model spatial distribution of proteins and RNAs during development, we considered a series of cells in a one-dimensional domain, representing the rostral-caudal axis of the embryo. These cells are under influence of varying strengths of RA and FGF determined by:


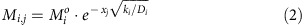


Where *M*_*i,j*_ is the strength of morphogen *i* at position *j*. 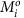 is the strength of morphogen *i* at the boundary of the domain where the morphogen is synthesized. *D*_*j*_ is the diffusion rate of the morphogen. *k*_*i*_ is the degradation rate of the morphogen. *x*_*j*_ is the distance between position *j* and the boundary. This approximates a reaction diffusion system for RA and FGF at steady state. In each simulation, we first ran the system to steady state without morphogen signals, and then we raised the morphogen strengths to particular values and continued the simulation. To consider temporal noise in the RA and FGF gradients, the values of RA and FGF are subject to random multiplicative perturbations during the simulation^59^. The perturbations of these parameters were introduced in each 1/ω time interval, where ω is the frequency of the noise (ω=10 day^*−*1^). In each time interval, random parameters were chosen for the morphogens in ranges proportional to their mean concentrations specified at the beginning of simulations.

### Robustness and accuracy of boundary formation

To evaluate the robustness of the Hoxa5/Hoxc8 boundary, we ran multiple simulations for the one-dimensional system and aligned them to create a two-dimensional space. Due to the noisy morphogen signals, the boundary between Hoxa5-expressing cells (defined as a cell expressing >0.5 unit of Hoxa5 protein) and Hoxc8-expressing cells were not always sharp. We defined the ‘transition zone' of a protein as the domain in which the percentages of cells expressing that protein at a particular horizontal position are between 15 and 85%, and we defined ‘transition width' (Ω) as the width of the transition zone. We assumed that the expression boundary with respect to the protein is at the midpoint of the transition zone. To evaluate the accuracy of the boundary position in terms of two proteins, we measured the distance between two expression boundaries.

### Topology selection

To search for a plausible signalling network controlling Hoxa5/Hoxc8 boundary formation, we first built a basal model without miRNA (Fig. 4a). The interactions of the basal model were based on previously-reported experimental data^10^. The parameter values of the model were chosen such that the model was able to reproduce the observed overlapping expression of Hoxa5 and Hoxc8 in the absence of miRNA (Fig. 3a). We considered six possible interactions involving a hypothetical miRNA named mir-x (Fig. 4a). We assumed that miRNA mainly inhibits the translational activity of its target mRNA. Therefore, each possible interaction can be quantified by a coefficient *ω*_*j*→*i*_ which can adopt two (for miRNA) or three (for protein) representative values: *ω*_*j*→*i*_=0, −1 or 1, where 0 means no interaction, −1 means inhibition and 1 means activation. This gave rise to 324 possible network topologies in total. We ran simulations with these topologies and ranked them based on the following metric:


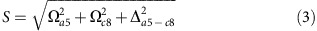


Where, Ω_*a*5_ is the transition width of Hoxa5. Ω_*a*8_ is the transition width of Hoxc8. Δ_*a*5−*c*8_ is the distance between the Hoxa5 expression boundary and the Hoxc8 expression boundary. A low score represents a robust Hoxa5/Hoxc8 boundary. We identified three consensus interactions based on the top 2% of topologies (Supplementary Fig. 5a). We built a mathematical model for mir-x based on these interactions (Fig. 4a). We tested these interactions experimentally in subsequent experiments. The parameter values of the basal and mir-x network are listed in Supplementary Table 2. To exclude the possibility that the search results were sensitive to the interaction strength, we repeated this procedure six times with different strengths of *ω*_*j*→*i*_ in a range of 0.2–1.2. The topology with the best performance was robust to the choice of interaction strength in the range of 0.4–1.2. Nonetheless, we cannot exclude the possibility that other network topologies may satisfy the constraints we imposed in this study.

### Simulation of mutant cells/embryo

When simulating *Dicer*^*−/−*^cells, we set the basal rate of mir-x production to be −100. When simulating cells with fast mir-x regulation, we set the relaxation rate of mir-x to be ten times its normal rate.

### Data availability

Gene expression microarray data during ESC-derived MN differentiation have been deposited in the Gene Expression Omnibus under accession code GSE91080, as well as being summarized in Supplementary Table 1. NanoString miRNA microarray expression data is included in this published article (Supplementary Table 3). The authors declare that all data supporting the findings of this study are available within the article and its Supplementary Information files or from the corresponding author upon reasonable request.

## Additional information

**How to cite this article:** Li, C.-J. *et al.* MicroRNA filters Hox temporal transcription noise to confer boundary formation in the spinal cord. *Nat. Commun.* **8,** 14685 doi: 10.1038/ncomms14685 (2017).

**Publisher's note:** Springer Nature remains neutral with regard to jurisdictional claims in published maps and institutional affiliations.

## Supplementary Material

Supplementary Information Supplementary Figures, Supplementary Tables.
