# Supplementary material for: MicroRNA filters Hox temporal transcription noise to confer boundary formation in the spinal cord
Source: Nat Commun. 2017 Mar 24;8:14685. doi: 10.1038/ncomms14685 (PMC5376671; doi:10.1038/ncomms14685)
Supplement: Supplementary Information — Supplementary Figures, Supplementary Tables. [file ncomms14685-s1.pdf]

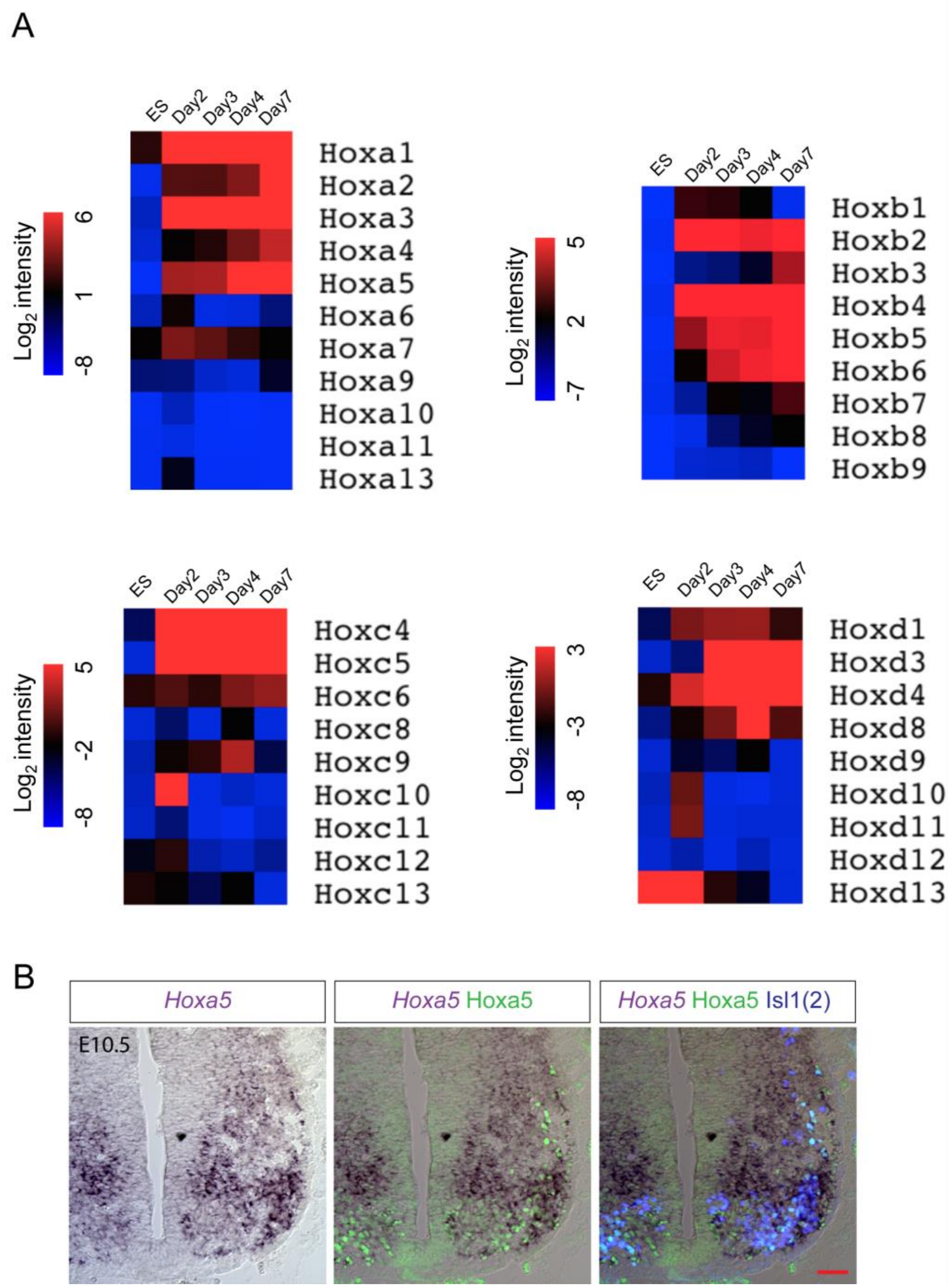

Supplementary Figure 1. Strong rostral *Hox1*–*Hox5* expression upon RA induction during motor neuron differentiation.

(A) Rapid induction of Hox1–Hox5 gene expression upon RA/SAG induction. Heat map represents fold-changes in gene expression along the four Hox clusters at five time-points during

motor neuron differentiation. (B) Expression of *Hoxa5* mRNA and protein distribution examined by *in situ* hybridization together with immunostaining on E10.5 spinal cord sections. VZ: progenitor ventricular zone; MZ: postmitotic marginal zone. Scale bar represents 50 µm.

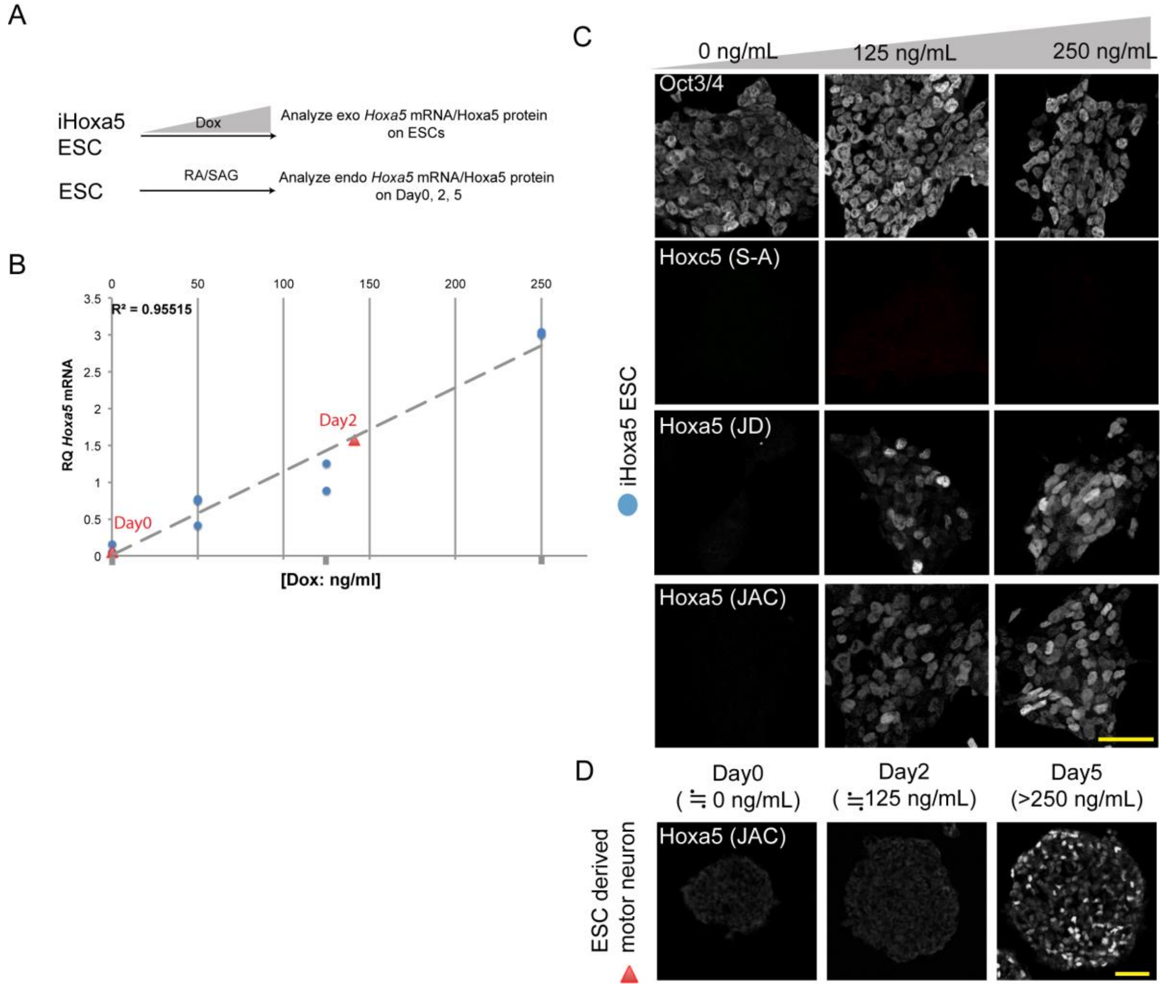

Supplementary Figure 2. Validation of the sensitivity and specificity of Hoxa5 antibody.

(A) Direct measurement and comparison of *Hoxa5* mRNA and protein from induced "Tet ON" iHoxa5 ESC and ESC-derived motor neurons. (B) Relative expression level of serial doxycycline (Dox) dosage treatment in iHoxa5ESCs. Blue dots indicate the induced exogenous *Hoxa5* level from 0 ~ 250 ng mL^{-1}. Red triangles show relative *Hoxa5* level of Day 0 (left) and Day 2 (right) EBs. (C) Robust exoHoxa5 protein expression is already detected for low-dose Dox [125 ng mL^{-1}], while the corresponding concentration of Dox

for endo*Hoxa5* in Day 2 control EBs did not manifest detectable Hoxa5 protein. Scale bar represents 50 μm. (D) Hoxa5 antibodies from JD: Jeremy Dasen, JAC: our in-house one. Hoxc5 (Sigma-Aldrich) is not detectable in all conditions from iHoxa5 after Dox treatment. Scale bar represents 50 μm.

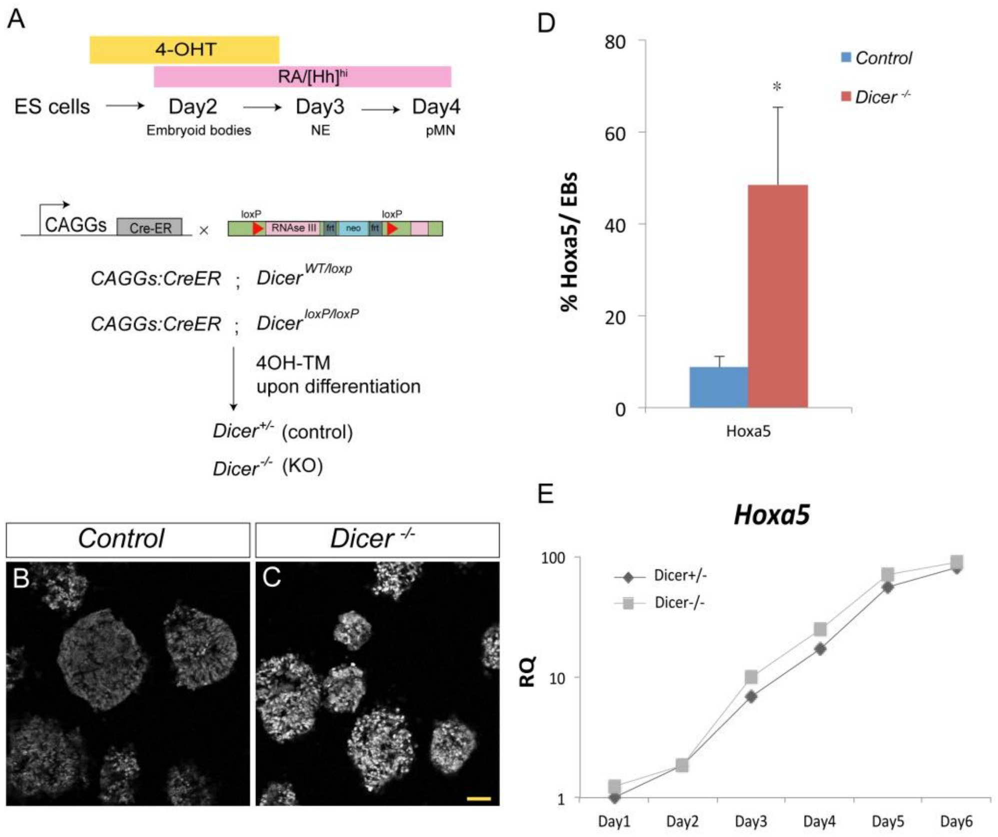

Supplementary Figure 3. Precocious Hoxa5 expression in the pMN in *Dicer*$^{-/-}$ embryoid bodies.

(A) *CAGGs:CreER*; *Dicer*$^{WT/loxp}$ and *CAGGs:CreER*; *Dicer* $^{loxp/loxp}$ ES cell lines (referred to as *Dicer*$^{+/-}$ and *Dicer*$^{-/-}$) after 4-hydroxytamoxifen (4-OHT) treatment. ESCs were differentiated with RA (1 μM) and SAG (500 nM) on Day 2, and spinal progenitor identities were determined by immunostaining embryoid body sections on Day 4. (B and C) Increase in the fraction of cells expressing Hoxa5 precociously in *Dicer*$^{-/-}$ EBs on Day 4. Scale bar represents 50 μm. (D) Quantification of Hoxa5^{on} progenitor cells (percentage of total cells, mean ± SD; n=3 independent experiments) reveals an increase

of Hoxa5 in pMN ($p < 0.01$) progenitors in *Dicer*$^{-/-}$ embryoid bodies under RA/SAG conditions. (E) qPCR analysis of *Hoxa5* during MN differentiation in control and *Dicer*$^{-/-}$ EBs. mRNA levels were normalized to that of ESCs (mean ± SD, n = 3 independent experiments).

Fig S4

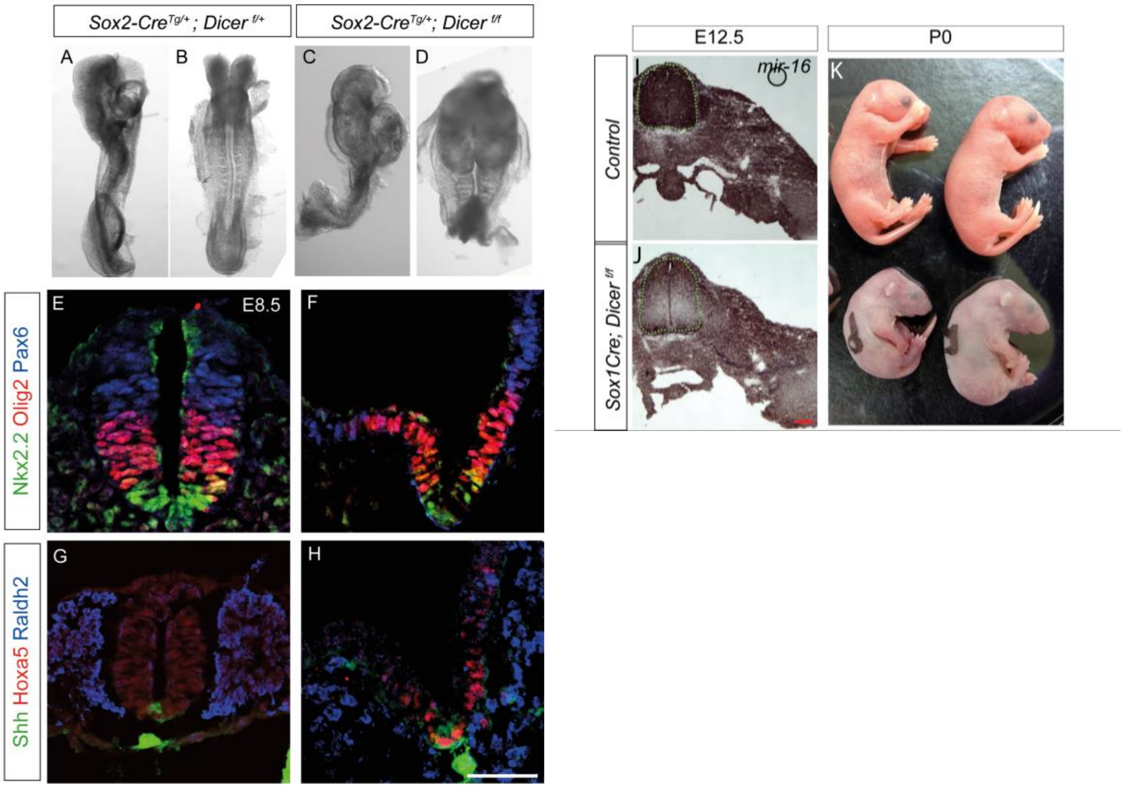

Supplementary Figure 4. Characterization of spinal phenotype in *Dicer* $^{epiblast\Delta}$ and *Dicer* $^{neural\Delta}$ embryos.

(A~D) Lateral (A and C) and dorsal (B and D) view of control (*Sox2-Cre*$^{Tg/+}$; *Dicer*$^{WT/loxp}$) and *Dicer* $^{epiblast\Delta}$ (*Sox2-Cre*$^{Tg/+}$; *Dicer*$^{loxp/loxp}$) embryos at E8.5. (E and F) *Dicer* $^{epiblast\Delta}$ embryos manifest robust Pax6 (p0~p2), Olig2 (pMN), and Nkx2.2 (p3) expression despite open neural tube defects. (G and H) Precocious Hoxa5 expression was detected in the pMN, whereas morphogens such as Shh and the RA-producing enzyme, Raldh2, were unaffected in the *Dicer* $^{epiblast\Delta}$ embryos. Scale bar represents 50 μm. (I and J) Spinal cord sections from E12.5 control and *Sox1*$^{Cre/+}$; *Dicer*$^{loxp/loxp}$ embryos were examined for *miR-16* expression by *in situ* hybridization. Scale bar represents 50 μm. (K) *Sox1*$^{Cre/+}$; *Dicer*$^{loxp/loxp}$ embryos die perinatally at P0 with a bent rostrocaudal axis and lowering of forelimbs, which is an indication of motor neuron death.

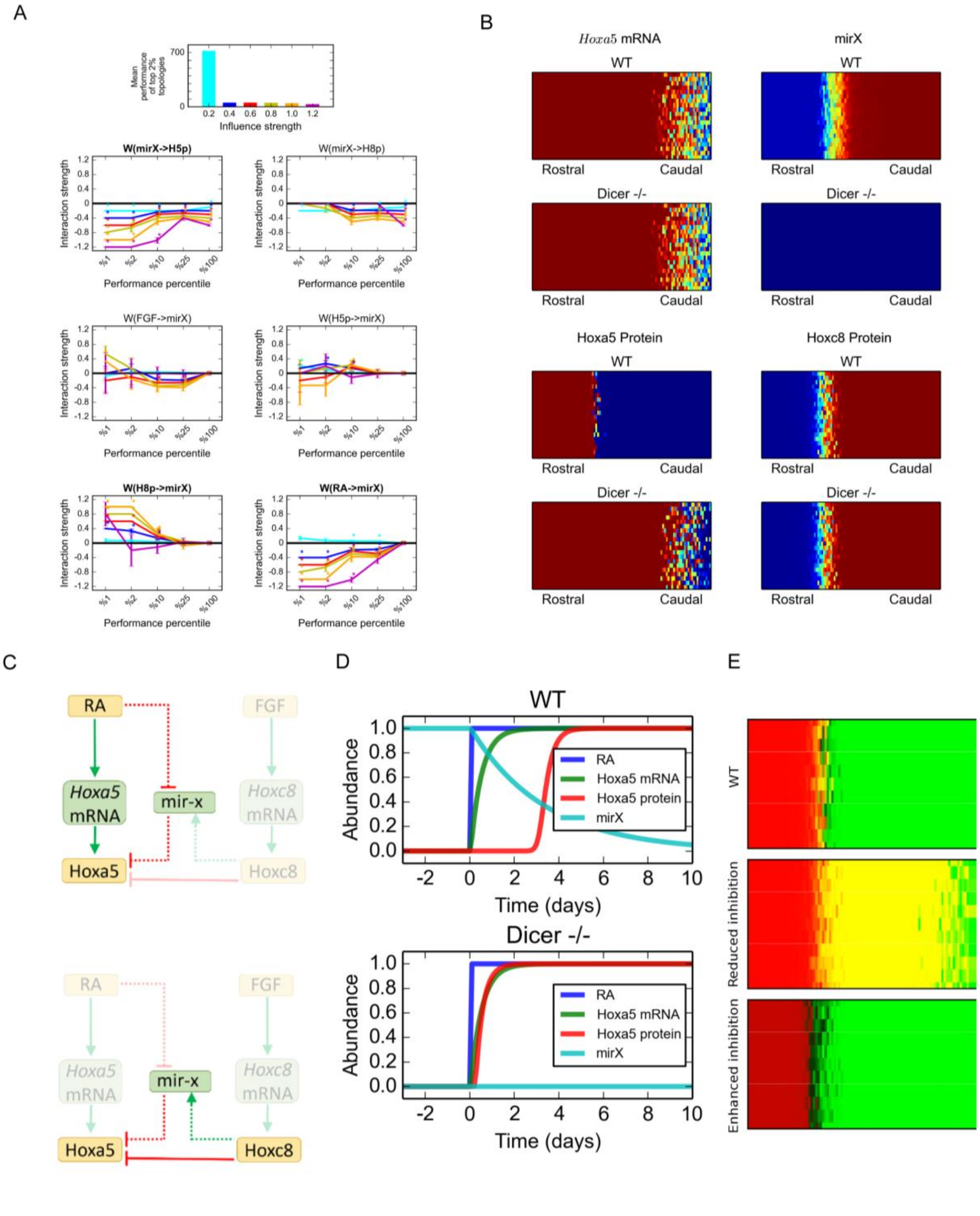

Supplementary Figure 5. Simulation of Hox-miRNA network in pMN.

(A) Top bar chart: mean performance scores for six strengths of interaction used for the topology search. Bottom panels: average interacting strengths of various percentiles of top scored topologies for six possible interactions. Interactions with purely positive or negative 95% confidence intervals are marked with an asterisk. Each line represents a search with a specific interaction strength. (B) Spatial distributions of four molecules at steady state. (C) Two coherent feed-forward loops in the predicted miRNA network. (D) Time-course simulations of a WT cell and a *Dicer*$^{-/-}$cell in response to RA stimulation. (E) Comparisons of the effect of removing Hoxc8->Hoxa5 inhibition and enhancing the Hoxc8->Hoxa5 inhibition. Steady-state expression levels of Hoxa5 (red) and Hoxc5 (green) are shown. For removal of inhibition, inhibition strengths were reduced to 1/10 of the original values. For enhancement, these parameters were increased 10-fold.

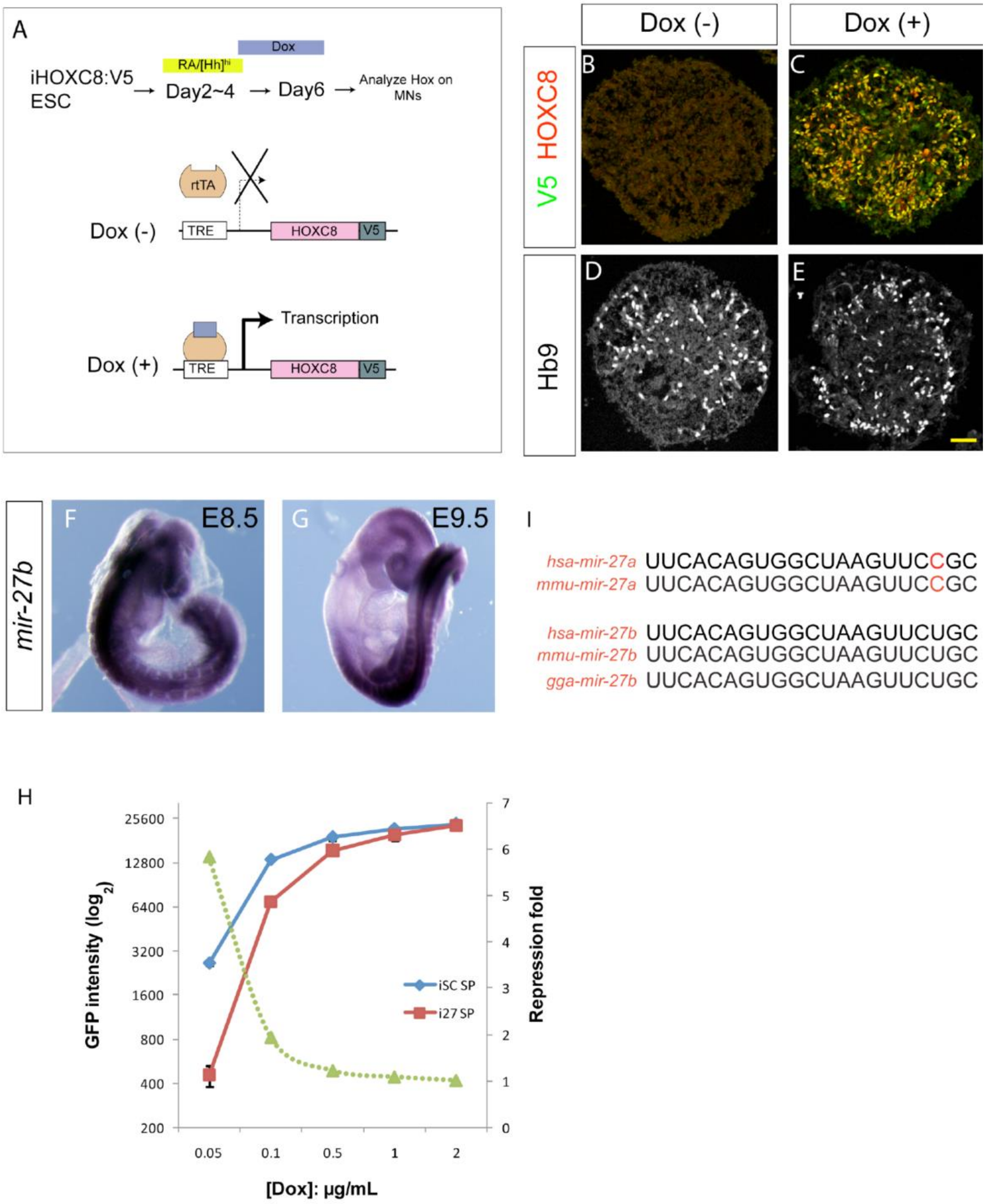

Supplementary Figure 6. ***mir-27b*** **is expressed in the caudal brachial spinal cord and maintained by Hoxc8.**

(A) The design of inducible “Tet-ON” ES cell lines expressing human HOXC8 under the doxycycline (Dox)-regulated promoter. In the presence of Dox, the reverse tTA (rtTA) activator is recruited to the TRE (Tetracycline Response Element), thereby initiating the transcription of the downstream gene. (B–E) Activated expression of exogenous HOXC8:V5 in inducible HOXC8 (iHOXC8) Day 7 embryoid bodies differentiated under RA/SAG conditions, with Dox treatment on Day 4. Generic postmitotic MN cell fate is not affected, as revealed by Hb9. Scale bar represents 50 μm. (F and G) Whole-mount *in situ* hybridization indicated that *mir-27b* is ubiquitously expressed at E8.5, then gradually caudalized to the Hoxc8 domain at E9.5. (H) Induced GFP expression is dependent on doxycycline concentration. GFP kinetics shows that the kinetics of doxycycline (Dox) penetration into ESCs is concentration-dependent. The sigmoidal curve is consistent with increasing doxycycline concentration. High doses of Dox (>0.5μg/mL) saturate the GFP intensity between scrambled and *mir-27* sponge ESCs (represented by blue and red lines, respectively), indicating that the sponge has decayed and saturated the endogenous *mir-27*. The activity of *miR-27* was calculated by determining the fold decrease between GFP mean fluorescence of control and *iMir27SP* ES cells (green line). (I) Comparison of miRNA sequence for *mir-27* between human, mouse, and chicken.

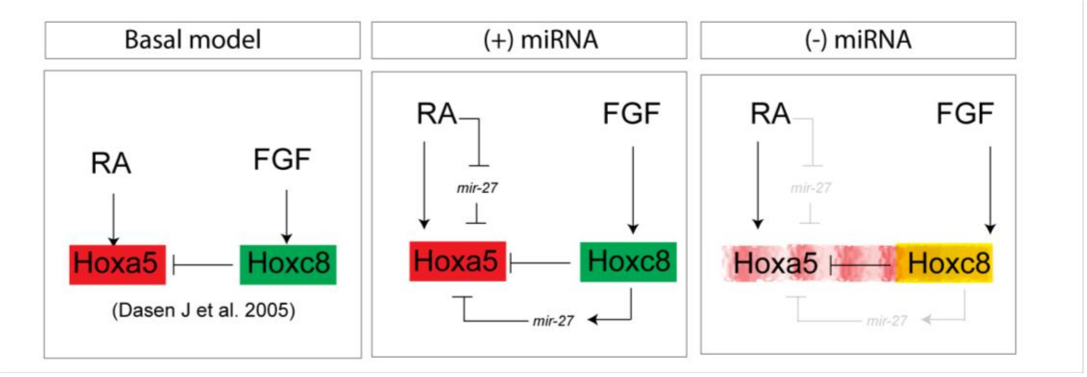

Supplementary Figure 7. Model of the role of *mir-27* in the control of Hoxa5 noise and the Hox boundary.

Previous studies suggest that RA induces Hoxa5, whereas FGF activates Hoxc8. We reveal that Hoxa5 transcription in progenitor cells fluctuates, and translation of fluctuating transcripts at this time propagates noise, leading to strong stochastic variability. We further underscore two critical, coherent forward loops involving *mir-27* that are capable of preventing precocious Hoxa5 protein expression and can maintain the critically sharp boundary between Hoxa5 and Hoxc8 protein expression in embryonic spinal cords.

SUPPLEMENTARY TABLE 1. **Microarray analyses of the expression of Hox clusters during ESC~MN differentiation.**

GeneSymbol	[ES-WT](raw)	[Day2-WT](raw)	[Day3-WT](raw)	[Day4-WT](raw)	[Day7-WT](raw)
Hoxa1	643.02	44800.65	32857.36	14887.99	11109.28
Hoxa1	7.82	147.51	87.28	48.02	181.95
Hoxa2	17.36	1043.51	1025.71	1483.69	3059.93
Hoxa3	56.41	3577.06	3593.41	3499.21	8992.94
Hoxa3	24.03	79.11	23.02	68.30	92.87
Hoxa4	41.37	284.26	584.73	1316.24	2118.15
Hoxa5	6.74	1799.29	1871.21	2690.28	12662.38
Hoxa6	60.83	385.56	25.26	31.81	120.57
Hoxa7	260.06	1438.43	1155.43	691.26	230.79
Hoxa9	125.37	121.20	47.81	33.08	193.41
Hoxa9	57.61	73.52	42.87	33.39	41.22
Hoxa10	7.95	61.17	5.31	2.54	6.65
Hoxa11	12.18	23.25	5.80	5.01	6.98
Hoxa11as	80.58	212.92	33.17	28.86	57.59
Hoxa13	11.39	205.82	8.22	8.76	6.42
Hoxa13	16.53	62.44	4.50	6.60	5.45
Hoxb1	9.41	3041.34	2712.78	1505.46	98.51
Hoxb2	47.94	9267.92	8093.18	7739.15	8138.97
Hoxb3	6.77	702.95	827.53	1269.44	5875.28
Hoxb3	46.73	1248.83	1665.76	1720.14	2877.88
Hoxb4	81.98	10324.65	11187.57	9874.21	20860.53
Hoxb5	27.91	5366.01	7687.94	7527.02	40840.50
Hoxb6	60.26	1708.35	6889.27	7783.21	24469.08
Hoxb7	102.19	594.30	1758.34	1431.33	3506.38
Hoxb8	7.02	121.88	873.38	1288.48	1512.43

Hoxb9	7.45	268.44	308.49	370.09	19.28
Hoxb13	183.70	240.80	168.75	201.67	10.96
Hoxc4	31.13	743.45	6718.86	6851.15	36725.43
Hoxc5	7.76	3145.96	1064.49	2167.29	7272.58
Hoxc6	157.80	269.40	167.23	386.93	455.47
Hoxc8	7.70	28.68	6.54	47.67	6.42
Hoxc9	13.73	86.18	172.20	525.79	33.54
Hoxc10	11.95	763.67	5.10	10.68	6.61
Hoxc11	10.33	25.87	5.15	2.98	9.39
Hoxc12	43.50	160.23	14.99	11.37	19.91
Hoxc13	131.43	64.68	32.84	53.20	6.44
Hoxd1	26.85	113.11	129.75	131.24	70.59
Hoxd3	8.19	22.25	315.09	603.85	5244.70
Hoxd4	58.34	166.32	1705.85	5424.50	2101.16
Hoxd8	19.48	52.63	110.33	194.98	88.20
Hoxd9	7.31	32.97	25.83	40.56	6.10
Hoxd10	10.84	102.35	4.70	2.93	6.06
Hoxd11	8.65	111.92	5.11	6.68	6.22
Hoxd11	7.28	10.21	4.69	2.16	6.04
Hoxd12	7.13	13.23	4.56	11.44	5.679093
Hoxd13	191.24	222.56	63.81	35.61	5.8786526

SUPPLEMENTARY TABLE 2. Parameter values of the mathematical model.

Parameter	Description	Value
γ_{H5m}	Timescale of Hoxa5 mRNA	2
γ_{H5p}	Timescale of Hoxa5 protein	3
γ_{H8m}	Timescale of Hoxc8 mRNA	3
γ_{H8p}	Timescale of Hoxc8 protein	1
γ_{mirX}	Timescale of mir-x	0.3
σ_{H5m}	Steepness of sigmoidal function for Hoxa5 mRNA	8
σ_{H5p}	Steepness of sigmoidal function for Hoxa5 protein	2
σ_{H8m}	Steepness of sigmoidal function for Hoxc8 mRNA	8
σ_{H8p}	Steepness of sigmoidal function for Hoxc8 protein	8
σ_{mirX}	Steepness of sigmoidal function for of mir-x	5
$\omega^{o}_{\mathrm{H5m}}$	Basal production rate of Hoxa5 mRNA	-2
$\omega^{o}_{\mathrm{H5p}}$	Basal production rate of Hoxa5 protein	-1
$\omega^{o}_{\mathrm{H8m}}$	Basal production rate of Hoxc8 mRNA	-0.9
$\omega^{o}_{\mathrm{H8p}}$	Basal production rate of Hoxc8 protein	-0.5
$\omega^{o}_{\mathrm{mirX}}$	Basal production rate of mir-x	2
$\omega_{\mathrm{RA}\to\mathrm{H5m}}$	Activation strength of RA on Hoxa5 mRNA	8
$\omega_{\mathrm{FGF}\to\mathrm{H8m}}$	Activation strength of RA on Hoxc8 mRNA	9
$\omega_{\mathrm{H5m}\to\mathrm{H5p}}$	Hoxa5 translational efficiency	3
$\omega_{\mathrm{H8m}\to\mathrm{H8p}}$	Hoxc8 translational efficiency	2
$\omega_{\mathrm{H8p}\to\mathrm{H5p}}$	Inhibition strength of Hoxc8 protein on Hoxa5 protein	-1
$\omega_{\mathrm{RA}\to\mathrm{mirX}}$	Inhibition strength of RA on mir-X	-1
$\omega_{\mathrm{mirX}\to\mathrm{H5p}}$	Inhibition strength of mir-X on Hoxa5 translation	-1
$\omega_{\mathrm{H8p}\to\mathrm{mirX}}$	Activation strength of H8p on mir-X	1

SUPPLEMENTARY TABLE 3. **NanoString miRNA profiling during ESC~MN differentiation.**

**miRNA**	**ES**	**D2**	**D3**	**D4**
mmu-miR-125a-5p	1	0.972817368	0.4035864	0.808777105
mmu-miR-150	1	0.464152353	0.416088766	0.416088766
mmu-miR-21	1	0.281845748	0.151285432	0.117521424
mmu-miR-27a	1	0.50658357	0.287996823	0.273347997
mmu-miR-27b	1	0.613472102	0.327551315	0.531078346
mmu-miR-291a-3p	1	0.825423717	0.191697187	0.068455498
mmu-miR-291a-5p	1	0.416731411	0.060456952	0.056111878
mmu-miR-291b-3p	1	0.774713004	0.161991145	0.152803354
mmu-miR-292-3p	1	0.728370385	0.166330989	0.04877503
mmu-miR-292-5p	1	0.428417702	0.063414488	0.058288123
mmu-miR-293	1	0.811089535	0.177254057	0.068231838
mmu-miR-294	1	0.730470624	0.175156488	0.066715819
mmu-miR-295	1	0.709266272	0.164904699	0.05387214
mmu-miR-297c	1	0.69676354	0.545112285	0.515191546
mmu-miR-29a	1	0.385681376	0.197275923	0.092623379
mmu-miR-29b	1	0.493242495	0.253229201	0.114633876
mmu-miR-302a	1	0.463025243	0.144307476	0.055104635
mmu-miR-302b	1	0.544991801	0.266466248	0.266466248
mmu-miR-302d	1	0.481155077	0.160025467	0.077335989
mmu-miR-367	1	0.632789306	0.149780004	0.059376094
mmu-miR-466a-3p+mmu-miR-466b-3-3p	1	0.659855082	0.447068021	0.346936888
mmu-miR-467a	1	0.754168511	0.575475874	0.575475874
mmu-miR-467c	1	0.780488836	0.706909675	0.472048376
mmu-miR-669a	1	0.709116127	0.620665742	0.491741268
mmu-miR-669f	1	0.605479058	0.478251354	0.302467515
mmu-miR-669o	1	0.5557482	0.520138704	0.520138704
mmu-miR-99b	1	0.998797056	0.551938862	0.595881687

All miRNAs that exhibit continuous downregulation from ESC to Day4.

SUPPLEMENTARY TABLE 4. **Primers and oligos used in this study.**

Primers for genotyping

**Gene**	**Forward Primer**	**Reverse Primer**
Dicer WT/loxp	CCTGACAGTGACGGTCCAAAG	CATGACTCTTCAACTCAAACT
Dicer Deletion	CCTGACAGTGACGGTCCAAAG	CCTGAGCAAGGCAAGTCATTC
GFP	CCCTGAAGTTCATCTGCACCAC	TTCTCGTTGGGGTCTTTGCTC
Cre	TGATGGACATGTTCAGGGATC	CAGCCACCAGCTTGCATGA
mir-27a WT	GGGAATGCTTCTTCCCTCTT	CACGACTTTGCTGTGGACCT
mir-27a Del	GGGAATGCTTCTTCCCTCTT	CTATCTGCTTTGGGGAACCA
mir-27b WT	CTCTGTGCTATGCCTCAGCTTAT	CCCCATCTCACCTTCTCTTCAG
mir-27b Del	CTCTGTGCTATGCCTCAGCTTAT	TCAGAAAGGCTCTACAGACAAGG

Primers for Luciferase 3'-UTR

**Name**	**Primer**
mut1-mir27F	AACTCGAGCCTCTTGTGATCATTGACACTAGAAGCCCTGTTCTCGT
mut2-mir27R	AAGCGGCCGCGAGTGTCATTTCGTCACAGAGCCACTAGCCACTACACGTTG
mut3-mir27R	AAGCGGCCGCAAGTGTCATTGCTTAAATATTCAGACTTGGACAATATTTGTAAC

Forward primer XhoI cutting site: CTCGAG
Reverse primer NotI cutting site: GCGGCCGC

Primers for primary miRNA sequence

**miRNA name**	**Assay ID**	**Mature miRNA Sequence**
mmu-miR-16	391	UAGCAGCACGUAAAUAUUGGCG
mmu-miR-27a	408	UUCACAGUGGCUAAGUUCCGC
mmu-miR-27b	409	UUCACAGUGGCUAAGUUCUGC

snoRNA234 (mouse)	1234	CTTTTGGAACTGAATCTAAGTGATTTAACAAAAATTCGTCACTACCACTGAGA
snoRNA202 (mouse)	1232	GCTGTACTGACTTGATGAAAGTACTTTTGAACCCTTTTCCATCTGATG

Primers for Q-PCR

Gene	Forward Primer	Reverse Primer
Hb9	GTTGGAGCTGGAACACCAGT	GCTCTTTGGCCTTTTTGCT
Olig2	AAGCCCGCTGTTTTCTTTCT	CGGAAGAGGTGGAAGGTTAG
Hoxa5	TGTACGTGGAAGTGTTCCTGTC	GTCACAGTTTTCGTCACAGAGC
Gapdh	TGACCACAGTCCATGCCATC	GACGGACACATTGGGGGTAG
Hprt	TCATGAAGGAGATGGGAGGC	CCACCAATAACTTTTATGTCCCC

Sequence for miRNA sponge

Mir-27b sponge (8 repeats, ccgg as spacer)
GCAGAACTTCGGACTGTGAAccggGCAGAACTTCGGACTGTGAAccggGCAGAACTTCGGACTGTGAAccggGCAGAACTTCGGACTGTGAAccggGCAGAACTTCGGACTGTGAAccggGCAGAACTTCGGACTGTGAAccggGCAGAACTTCGGACTGTGAAccggGCAGAACTTCGGACTGTGAA

Mir-scramble sponge (8 repeat, ccgg as spacer)
5'
TTCACAATGCGTTATCGGATGTccggTTCACAATGCGTTATCGGATGTccggTTCACAATGCGTTATCGGATGTccggTTCACAATGCGTTATCGGATGTccggTTCACAATGCGTTATCGGATGTccggTTCACAATGCGTTATCGGATGTccggTTCACAATGCGTTATCGGATGTccggTTCACAATGCGTTATCGGATGT

Oligos to make gRNA for in vitro transcription

Gene	T7 promoter		Target sequence	Scaffold sequence
23a-8-L (62mer)	TAATACGACTCACTATAGGG	GG	GAAAGAAGAGACAGTGAGAT	GTTTTAGAGCTAGAAATAGC
23a-8-R (62mer)	TAATACGACTCACTATAGGG	GG	GTGCACTGGCTCAGTTCAGC	GTTTTAGAGCTAGAAATAGC

23b-13-L (61mer)	TAATACGACTCACTATAGGG	G	GGAGAACAGGGTGTGTCCCA	GTTTTAGAGCTAGAAATAGC
23b-13-R (62mer)	TAATACGACTCACTATAGGG	GG	GCCCTTCATCTTCTTCTCCG	GTTTTAGAGCTAGAAATAGC
